# Mapping the hepatic neural niche: from three-dimensional innervation architecture to periportal interoception and human translation

**DOI:** 10.3389/fphys.2026.1955737

**Published:** 2026-10-06

**Authors:** Kun Wang, Xiangkun Mei, Hongqiang Gao

**Affiliations:** Department of General Medicine, Ganmei Hospital Affiliated to Kunming Medical University, Kunming, China

**Keywords:** hepatic neural niche, human translation, liver innervation, MASLD, periportal interoception, sensory afferents, spatial transcriptomics, three-dimensional imaging

## Abstract

The liver has traditionally been viewed as an organ regulated by circulating hormones and long-range autonomic reflexes, with its intrahepatic neural architecture receiving comparatively little systematic attention. However, recent advances in tissue clearing, three-dimensional (3D) volumetric imaging, neural tracing, and spatial omics are reshaping this view. Here we synthesize emerging evidence that the hepatic nervous system is organized around spatially defined neurovascular–cellular niches, and propose the periportal region as a dominant anatomical site for interoceptive integration, while acknowledging contributions from other hepatic compartments. We introduce a conceptual framework built on three spatial axes—hilum-to-periphery, portal-to-central, and neurovascular–cellular proximity—to replace the traditional binary distinction between portal tract and parenchymal innervation. We critically examine why conventional two-dimensional histology has systematically underestimated the complexity of hepatic innervation and evaluate how 3D imaging technologies are resolving long-standing controversies, particularly regarding the existence and extent of parasympathetic parenchymal innervation. We further review how chronic liver diseases, including metabolic dysfunction-associated steatohepatitis, fibrosis, and cholestasis, remodel the neural niche through spatially defined degenerative and regenerative processes. A dedicated cross-species analysis identifies fundamental anatomical, methodological, and interpretive gaps that prevent direct extrapolation of rodent findings to human hepatic neurobiology. Finally, we propose a translational roadmap toward a standardized human hepatic neural atlas that integrates 3D structural mapping with molecular identity and functional validation. This synthesis argues that understanding the liver as a spatially organized neural organ is essential for the rational design of neuromodulatory therapies targeting metabolic, inflammatory, and fibrotic liver disease, and it outlines how this framework could inform neural biomarkers, disease classification, and sex-aware precision medicine.

## 1 Introduction

### 1.1 The liver as a spatially organized neural organ

The liver has long been recognized as a target of autonomic nervous system regulation. Classical physiological studies established that sympathetic activation promotes hepatic glucose output and lipolysis, while parasympathetic (vagal) signaling modulates glycogen synthesis, bile secretion, and postprandial nutrient handling (Mizuno and Ueno, 2017; Borgmann and Fenselau, 2024). These regulatory effects were traditionally conceptualized as occurring through long-range autonomic reflexes: the brain integrates peripheral signals and returns efferent commands to the liver via the splanchnic and vagus nerves, with the intrahepatic neural fibers serving as passive transmission cables.

This view is now being challenged by evidence from multiple laboratories using modern imaging and tracing technologies. Five observations are particularly consequential. First, intrahepatic neural distribution is markedly spatially heterogeneous: nerve fiber density in the portal tract is orders of magnitude higher than in the hepatic lobule, and fiber density declines along a reproducible portal-to-central gradient (Jungermann and Kietzmann, 1996; Berthoud and Neuhuber, 2000). Second, nerve fibers do not enter the liver diffusely but follow defined anatomical routes—principally along the hepatic artery, portal vein, and bile ducts within the Glissonian sheath—before branching into finer periportal and, in some species, intralobular territories (Akiyoshi et al., 1998; Xu et al., 2026). Third, nerve terminals in the liver do not operate in isolation; rather, they form local microanatomical complexes with vascular endothelial cells, bile duct epithelial cells (cholangiocytes), hepatic stellate cells (HSCs), Kupffer cells, and components of the extracellular matrix, constituting what we term here the “hepatic neural niche” (McCuskey, 2004; Jensen et al., 2013). Fourth, the liver is not merely an efferent target: dense sensory (afferent) innervation, originating from both vagal nodose ganglia and spinal dorsal root ganglia (DRG), continuously transmits information about hepatic metabolic, osmotic, and inflammatory status to the central nervous system (Berthoud et al., 2024; Hwang et al., 2025). Fifth, high-resolution 3D imaging has revealed that the spatial relationship between nerves and their cellular partners is dynamic, undergoing substantial remodeling during metabolic and fibrotic liver disease (Adori et al., 2021; Liu et al., 2021).

These findings collectively suggest that hepatic neural function cannot be understood without first understanding hepatic neural architecture—where the nerves are, which cells they contact, and how these spatial relationships change in disease. To anchor these relationships, we propose an integrative conceptual figure (**Figure 1**) that maps the three spatial axes, the periportal interoceptive integration site, and disease-associated remodeling onto a single schematic.

**Figure 1:**
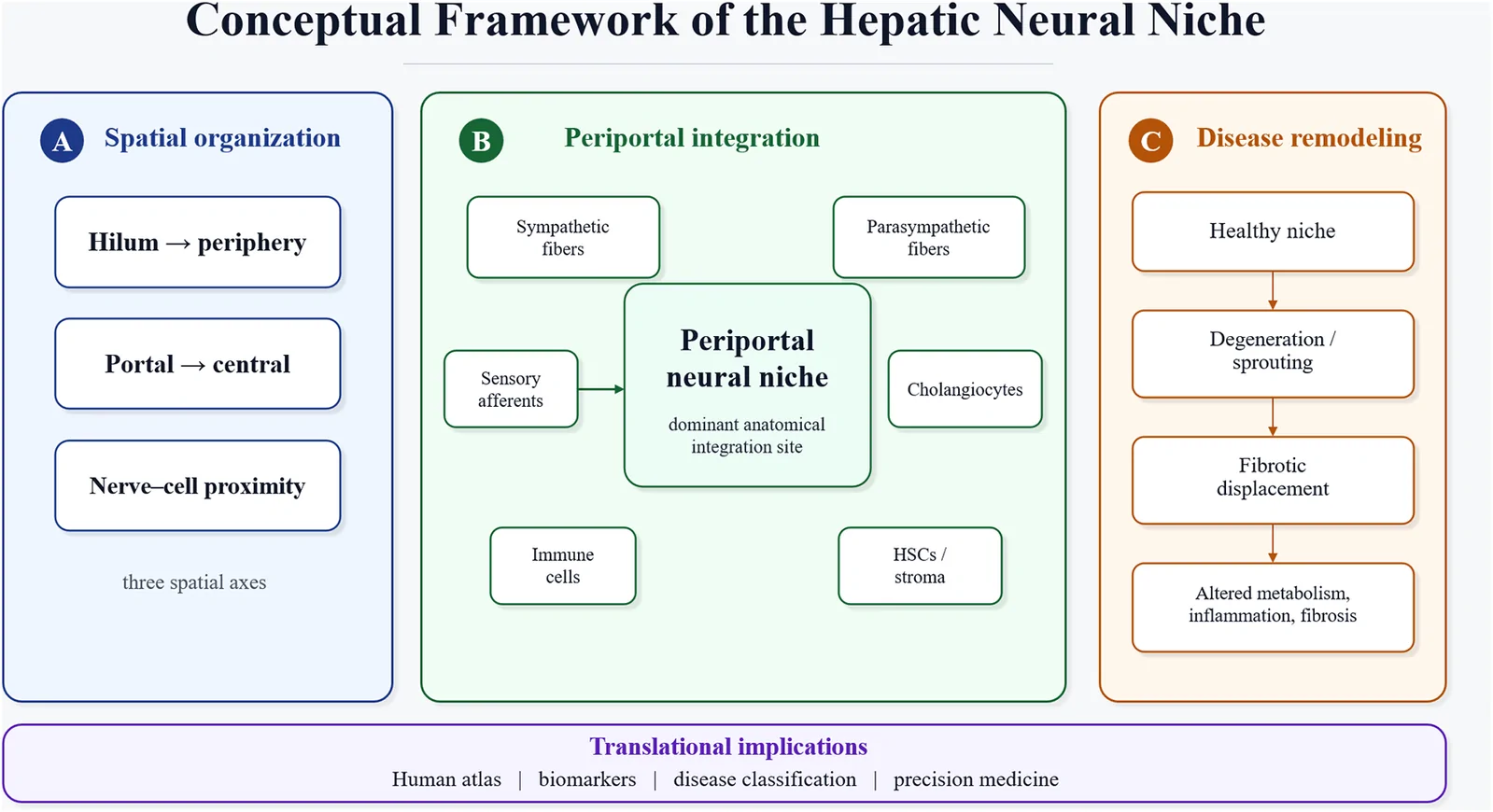
Conceptual framework of the hepatic neural niche. The hepatic neural niche is conceptualized as a spatially organized and dynamically remodeled neurocellular microenvironment. **(A)** Hepatic innervation can be considered along three complementary spatial axes, including hilum-to-periphery distribution, portal-to-central lobular organization, and local nerve–cell proximity. **(B)** The periportal region is proposed as a major anatomical integration site where sympathetic and parasympathetic fibers, sensory afferents, cholangiocytes, hepatic stellate cells (HSCs)/stromal cells, and immune cells converge to coordinate neural, metabolic, and immune signals. **(C)** During chronic liver injury, this niche may undergo neural degeneration and/or sprouting, followed by spatial displacement associated with fibrosis and broader alterations in metabolism, inflammation, and tissue remodeling. Defining these spatial and cellular relationships may facilitate the development of human hepatic neural atlases, biomarkers, disease classification frameworks, and precision therapeutic strategies. The scheme represents a conceptual model rather than an established linear causal pathway in human liver disease.

### 1.2 Why conventional two-dimensional anatomy is insufficient

The vast majority of our knowledge about liver innervation derives from thin (5–20 μm) histological sections analyzed by conventional immunohistochemistry. While this approach has been generative, it carries inherent limitations that have produced several persistent ambiguities in the field.

First, 2D sections cannot trace continuous nerve fibers. A single unmyelinated sympathetic axon may extend hundreds of microns through the liver parenchyma with multiple branch points; on any given 5 μm section, only a fragment of this fiber is visible, making it impossible to reconstruct complete axonal arbors or quantify total fiber length (Miller et al., 2021; Xu et al., 2026). Second, distinguishing genuine intraparenchymal nerve fibers from those associated with portal tract structures that appear in an oblique plane of section is notoriously difficult in 2D; a nerve bundle that appears to lie among hepatocytes may in fact be following a small portal venule or bile ductule that is not visible in the plane of section (Chen et al., 2024). Third, establishing whether a nerve terminal makes functional contact with a hepatocyte, cholangiocyte, or immune cell requires demonstrating sustained spatial proximity in three dimensions—a single 2D image showing a TH-positive varicosity adjacent to a hepatocyte is insufficient to distinguish a genuine neuro-effector junction from a chance cross-sectional juxtaposition (Chen et al., 2024; Lee et al., 2025). Fourth, 2D approaches preclude accurate quantification of fundamental neuroanatomical parameters, including total nerve fiber volume, length, branch-point density, nerve-to-vessel distance distributions, and the proportion of periportal versus intralobular fibers.

These limitations are not merely technical; they have generated substantive disagreements about whether human hepatocytes receive direct sympathetic or parasympathetic innervation, whether cholangiocytes are innervated by sensory fibers in health, and whether nerve loss in cirrhosis represents true degeneration or merely physical displacement by fibrotic tissue.

### 1.3 Scope and central questions

This review addresses four central questions that, we argue, must be answered before liver-directed neuromodulation can become a clinical reality:

1. How are hepatic nerves distributed in three-dimensional space? We synthesize evidence from tissue clearing, light-sheet microscopy, and super-resolution imaging to describe the 3D architecture of hepatic sympathetic, parasympathetic, and sensory innervation.
2. Why is the periportal region a dominant interoceptive integration site of the liver? We propose that the convergence of portal blood flow, dense neurovascular innervation, bile duct epithelium, and resident immune cells makes the periportal niche the liver’s dominant anatomical site for sensing and integrating metabolic, mechanical, and inflammatory signals, while recognizing that the hepatic capsule, sinusoidal regions, and metabolic zones also contribute to hepatic interoception.
3. How does chronic liver injury remodel the hepatic neural niche? We examine spatially defined structural changes—nerve degeneration, axonal sprouting, fibrotic entrapment, and potential reinnervation—across metabolic, fibrotic, and cholestatic liver diseases.
4. Why cannot rodent findings be directly extrapolated to humans? We provide a systematic cross-species comparison of hepatic innervation patterns, identify methodological confounds in rodent studies, and delineate the specific limitations of current human evidence.

We do not aim to comprehensively review the functional metabolic consequences of hepatic nerves, which have been covered in excellent recent reviews (Mizuno and Ueno, 2017; Berthoud et al., 2024; Borgmann and Fenselau, 2024). Rather, we focus on the structural and spatial organization of the hepatic neural niche—the anatomical foundation upon which all functional studies must rest.

### 1.4 Literature search and evidence selection

This is a narrative review, and a concise search strategy is reported here for transparency rather than to meet systematic-review standards. We searched PubMed, Web of Science, and Google Scholar for English-language articles published up to August 2026, using combinations of terms including “hepatic innervation”, “liver nerve”, “vagus nerve liver”, “sympathetic nerve liver”, “hepatic interoception”, “periportal”, “tissue clearing liver”, “spatial transcriptomics liver”, “metabolic dysfunction-associated steatotic liver disease”, “MASLD”, “MASH”, and “sex differences autonomic”. We prioritized primary studies—especially those employing tissue clearing, volumetric imaging, neural tracing, or human liver specimens—over secondary reviews, and weighted direct human evidence more heavily when evaluating translational claims. Where primary evidence was limited, inferences and hypotheses were flagged explicitly. Reference lists of included articles were screened for additional relevant studies. The final literature search was completed on 31 August 2026.

## 2 Defining the hepatic neural niche

### 2.1 Conceptual definition

We define the hepatic neural niche as a spatially organized functional unit composed of hepatic afferent and efferent nerve fibers, vascular structures (portal vein, hepatic artery, sinusoids), bile ducts, resident mesenchymal cells (HSCs, portal fibroblasts), immune cells (Kupffer cells, monocyte-derived macrophages), and the extracellular matrix, which collectively senses, integrates, and regulates local metabolic, hemodynamic, biliary, and inflammatory signals.

This definition departs from conventional descriptions of “liver innervation” in three respects. First, it explicitly includes non-neural cellular components as integral elements of the signaling unit, rather than passive recipients of neural input. Second, it emphasizes spatial organization: the composition, function, and disease vulnerability of the niche vary systematically along defined anatomical axes (Section 2.4). Third, it incorporates bidirectional signaling—the niche both receives efferent commands and generates afferent sensory output—making it a site of interoceptive integration rather than merely an effector target.

Relative to established concepts—neurovascular interactions, neuroimmune communication, and hepatic microenvironmental signaling—the hepatic neural niche is distinguished not by any single novel molecule or cell type but by three conceptual commitments that these earlier frameworks did not make jointly. First, it is a functional organizational unit: the nerve fiber, its vascular and mesenchymal scaffold, and its effector cells are treated as one signaling unit whose physiological output cannot be attributed to any component in isolation. Second, it is spatially explicit: because neural function is organized along hilum-to-periphery and portal-to-central gradients and depends on micrometer-scale nerve-to-cell proximity, spatial position is elevated from a descriptive detail to a primary organizing variable. Third, it is bidirectional: the niche both executes efferent autonomic commands and originates afferent interoceptive information, extending classical descriptions of liver innervation—which cast the liver primarily as an effector target—into a model of the liver as a site of sensory integration. We therefore offer the hepatic neural niche as a proposed analytical framework rather than as a claim of a newly discovered cell type.

### 2.2 Neural components

The hepatic sympathetic (noradrenergic) innervation—the quantitatively dominant input to the liver—originates primarily from preganglionic neurons in the intermediolateral cell column of the thoracic spinal cord (T7–T12), which synapse onto postganglionic neurons in the celiac and superior mesenteric ganglia (Strack et al., 1989). Postganglionic noradrenergic fibers reach the liver via the splanchnic nerves and enter the hepatic parenchyma along branches of the hepatic artery, portal vein, and bile ducts within the Glissonian sheath (Lin et al., 1995; Akiyoshi et al., 1998). Within the liver, tyrosine hydroxylase (TH)-positive sympathetic fibers form dense perivascular plexuses around portal vessels and extend into the portal space, where they associate with bile ducts and portal fibroblasts. Whether these fibers extend into the hepatic lobule to form close appositions with hepatocytes remains a species-specific question addressed in detail in Sections 4.2 and 7.

The preganglionic parasympathetic (vagal) innervation of the liver arises from cholinergic neurons in the dorsal motor nucleus of the vagus (DMV) in the brainstem, with axons traveling in the cervical and thoracic vagus nerve before reaching the liver via the common hepatic branch (Hwang et al., 2024). The anatomical fate of these fibers within the liver—specifically, whether they form postganglionic cholinergic terminals in the hepatic parenchyma—is the subject of considerable controversy (Section 4.3). In some species, intrahepatic ganglia have been described in the portal hilum, suggesting that at least some vagal preganglionic fibers may synapse locally rather than directly innervating hepatic parenchymal cells (McCuskey, 2004).

Spinal sensory afferent fibers have cell bodies that reside in thoracic DRG (T7–T13) and send peripheral processes to the liver via the splanchnic nerves and central processes to the spinal dorsal horn (Spencer et al., 2018; Berthoud et al., 2024). These fibers are predominantly peptidergic, expressing calcitonin gene-related peptide (CGRP), substance P, and the capsaicin receptor TRPV1, and are generally considered to convey nociceptive, inflammatory, and mechanical signals from the hepatic parenchyma and capsule.

The cell bodies of vagal sensory afferent neurons innervating the liver are located in the nodose (inferior vagal) ganglia, with central projections to the nucleus of the solitary tract (NTS) and area postrema (Berthoud and Powley, 1993; Hwang et al., 2025). These neurons are molecularly heterogeneous and respond to a broad range of metabolic, osmotic, and hormonal signals in the portal and hepatic microenvironment.

Beyond classical noradrenergic and cholinergic markers, subsets of hepatic nerve fibers express neuropeptides, including neuropeptide Y (NPY, largely co-localized with TH in sympathetic fibers), CGRP, substance P, galanin, and vasoactive intestinal peptide (VIP) (Ding et al., 1994; Akiyoshi et al., 1998). The spatial distribution of these peptidergic fibers is not uniform; NPY-positive sympathetic fibers are enriched in the periportal region, while CGRP- and substance P-positive sensory fibers are found around bile ducts, portal vessels, and in the hepatic capsule.

### 2.3 Non-neural components

The hepatic neural niche includes several non-neural cell types that are integral to its function:

Cholangiocytes, the bile duct epithelial cells, express receptors for acetylcholine (M3 muscarinic), norepinephrine (α1, β-adrenergic), and multiple neuropeptides including secretin, somatostatin, and substance P (Alvaro et al., 1997; Ehrlich et al., 2018). They are in direct anatomical proximity to both sympathetic and sensory nerve terminals in the portal space and actively secrete neurotrophic factors, including nerve growth factor (NGF), that regulate local nerve fiber density (Gigliozzi et al., 2004).

The endothelium of the portal vein and hepatic artery is densely innervated by sympathetic vasoconstrictor fibers and is a primary site of sensory afferent termination (Berthoud et al., 2024; Hwang et al., 2025). Endothelial cells in these vessels express mechanosensitive channels and respond to shear stress and transmural pressure changes, generating signals that are detected by adjacent sensory nerve terminals.

Kupffer cells and other monocyte-derived macrophages are liver-resident macrophages that express adrenergic and cholinergic receptors and are located in the sinusoidal lumen, where they can interact with nerve terminals in the space of Disse. Norepinephrine released from sympathetic terminals modulates Kupffer cell cytokine production. The cholinergic anti-inflammatory pathway, in which vagal signaling suppresses macrophage activation via α7 nicotinic receptors, has been characterized largely at the systemic level (Borovikova et al., 2000; Tracey, 2002); whether it operates through direct hepatic vagal innervation of Kupffer cells remains unestablished.

Hepatic stellate cells (HSCs) occupy the space of Disse and are positioned between sinusoidal endothelial cells and hepatocytes, making them anatomically poised to receive signals from intralobular nerve terminals. Myofibroblastic HSCs respond to acetylcholine with increased proliferation and collagen gene expression (Oben et al., 2003). HSCs also express neurotrophin receptors, including p75NTR, and participate in neurotrophin-dependent remodeling through factors such as NGF and BDNF (Passino et al., 2007). The extent to which local adrenergic signaling forms a self-sustaining HSC–nerve feedback loop in human liver disease remains unresolved.

Whether hepatocytes receive direct synaptic or parasynaptic neural input varies by species (Section 7). Even in the absence of direct contact, hepatocytes are exposed to neurotransmitters (norepinephrine, acetylcholine) that reach them via paracrine diffusion from periportal nerve terminals or via the systemic circulation. Hepatocytes express adrenergic and muscarinic receptors and respond to neural signals by modulating glucose output, lipid synthesis, and bile acid production (Shimazu, 1996).

The extracellular matrix—comprising collagen fibers and other components of the portal tract and space of Disse—provides structural support for nerve fibers and influences their trajectory, branching, and survival. In fibrosis, excessive collagen deposition physically separates nerve terminals from their cellular targets and may create mechanical barriers to axonal regeneration (Liu et al., 2021; Mizuno et al., 2021).

### 2.4 Three spatial dimensions

To move beyond the binary “portal tract vs. parenchyma” classification, we propose that the hepatic neural niche should be analyzed along three orthogonal spatial axes:

1. Hilum-to-periphery axis. Nerve bundles enter the liver at the hepatic hilum and branch progressively as they travel toward the liver capsule. The caliber, density, and composition of nerve trunks change along this axis: large mixed nerve bundles at the hilum give rise to smaller, predominantly perivascular branches in the lobar and segmental portal tracts, which in turn generate fine terminal fibers in the peripheral portal spaces and, in some species, the lobular parenchyma (Akiyoshi et al., 1998; Xu et al., 2026).
2. Portal-to-central axis. Within each hepatic lobule, nerve fiber density is highest in the portal tract and declines sharply toward the central vein. This gradient is steepest for sympathetic (TH-positive) fibers and reflects both the entry route of nerves (via portal structures) and the fact that the periportal hepatocytes (zone 1) are the first to encounter portal blood and are metabolically most active in gluconeogenesis and fatty acid oxidation—processes under sympathetic control (Jungermann and Kietzmann, 1996; Berthoud and Neuhuber, 2000).
3. Neurovascular–cellular proximity axis. At the micrometer scale, nerve terminals exhibit defined spatial relationships with specific non-neural cell types. Quantitative 3D analyses can measure the distance between individual nerve varicosities and the nearest cholangiocyte, Kupffer cell, HSC, or hepatocyte, providing a structural basis for inferring functional connectivity. This axis is dynamic: in steatohepatitis, the mean nerve-to-hepatocyte distance may increase due to nerve degeneration, while in early fibrosis, nerve-to-HSC distance may decrease due to HSC proliferation around nerve remnants (Adori et al., 2021; Liu et al., 2021; Mizuno et al., 2021).

This three-axis framework is not merely descriptive; it provides a quantitative language for comparing innervation patterns across species, experimental conditions, and disease states, and it identifies specific spatial parameters that must be reported in any future hepatic neural atlas (Section 3.5).

Critically, this spatial architecture is not a passive anatomical backdrop but a determinant of physiological function. Three spatial parameters—nerve density, nerve-to-cell proximity, and neural remodeling—translate directly into functional outcomes. Nerve density sets the capacity of the neural system to modulate a given metabolic zone: the high sympathetic fiber density in the periportal region aligns with the concentration of gluconeogenic and fatty-acid-oxidizing hepatocytes there, whereas the sparse innervation of pericentral (zone 3) hepatocytes implies a greater reliance on paracrine and endocrine signaling. Nerve-to-cell proximity determines whether transmission occurs through structured apposition or through volume transmission, and thus the spatial range over which a given neurotransmitter—norepinephrine, acetylcholine, or a neuropeptide—acts on glucose metabolism, lipid handling, bile secretion, or immune regulation. Neural remodeling couples disease to dysfunction: the progressive degeneration of sympathetic fibers in MASLD removes an anti-inflammatory and insulin-sensitizing brake, while aberrant sprouting and fibrotic displacement of nerves alter the effective connectivity between nerves and their targets. Together, these parameters define the mechanistic chain—spatial neural architecture, cellular signaling, physiological response, and disease phenotype—that the remainder of this review develops. This anatomy-to-physiology chain is summarized in **Figure 2**, which traces representative pathways from nerve subtype through target-cell signaling to physiological output and indicates the level of supporting evidence at each step.

**Figure 2:**
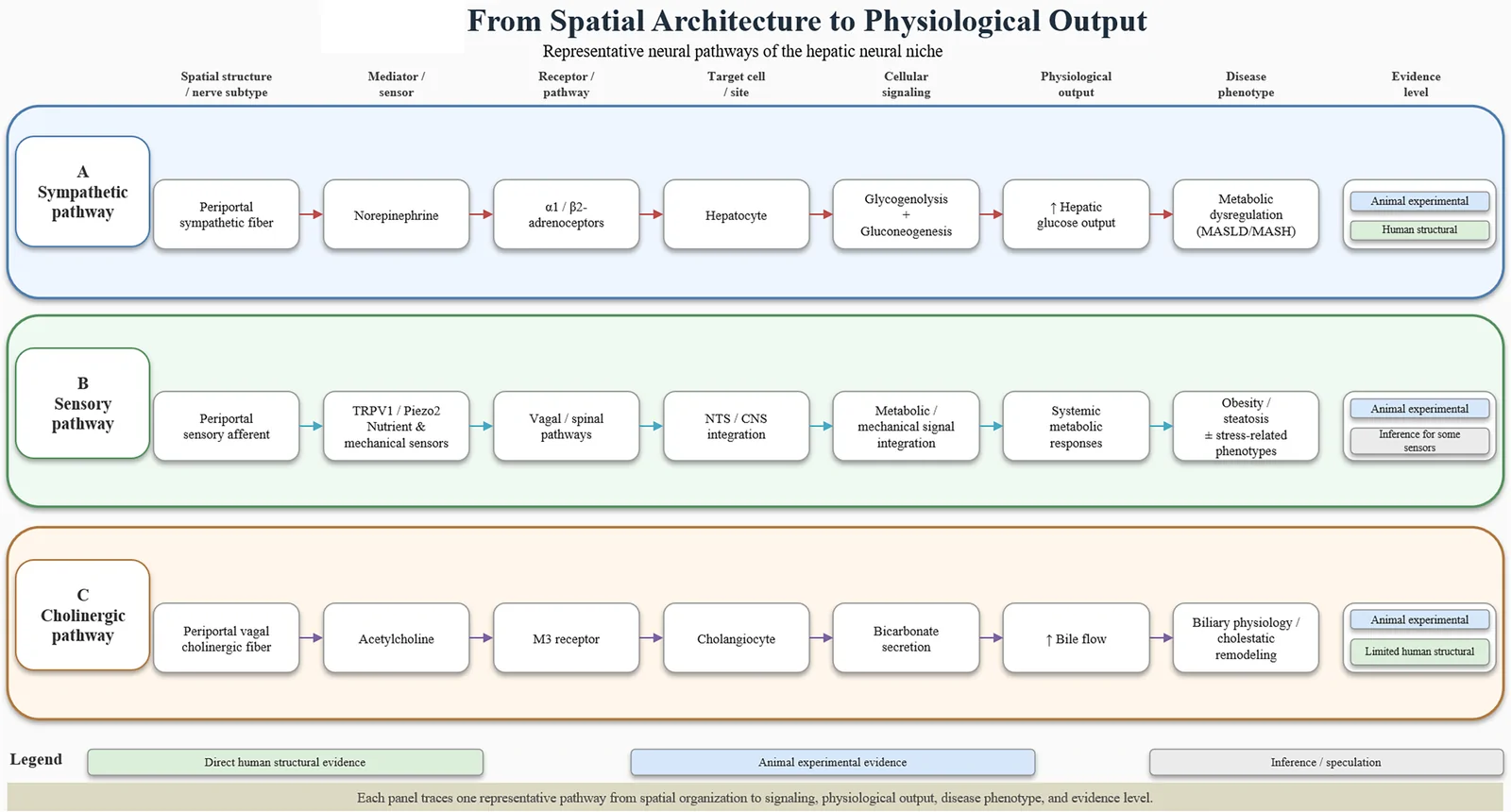
From spatial architecture to physiological output: representative neural pathways of the hepatic neural niche. The schematic illustrates three representative pathways linking the spatial organization of hepatic innervation to cellular signaling, physiological output, disease phenotype, and the corresponding level of evidence. **(A)** Periportal sympathetic fibers release norepinephrine, which acts on hepatocyte α1- and β2-adrenoceptors to promote glycogenolysis and gluconeogenesis, thereby increasing hepatic glucose output and potentially contributing to metabolic dysregulation. **(B)** Periportal sensory afferents detect nutrient and mechanical cues through sensors including TRPV1 and Piezo2 and relay these signals through vagal and spinal pathways to central integration sites, thereby influencing systemic metabolic responses and related disease phenotypes. **(C)** Periportal vagal cholinergic fibers release acetylcholine, which acts on M3 receptors on cholangiocytes to stimulate bicarbonate secretion and bile flow, linking cholinergic signaling to biliary physiology and cholestatic remodeling. Evidence levels are visually distinguished as direct human structural evidence, animal experimental evidence, or inference/speculation. The pathways shown are illustrative rather than exhaustive and are intended to integrate anatomical, mechanistic, physiological, and translational evidence across the hepatic neural niche.

## 3 Technologies for mapping hepatic innervation in three dimensions

### 3.1 Conventional histology and immunohistochemistry

Standard immunohistochemical markers remain the foundation of hepatic neuroanatomy. Protein gene product 9.5 (PGP9.5/UCHL1) and βIII-tubulin (TUBB3) serve as pan-neuronal markers that label all nerve fibers regardless of functional subtype (Lin et al., 1995; Akiyoshi et al., 1998). Tyrosine hydroxylase (TH) identifies catecholaminergic (sympathetic) fibers, while dopamine β-hydroxylase (DBH) provides an alternative noradrenergic marker with somewhat different sensitivity (Ding et al., 1994; Lin et al., 1995; Akiyoshi et al., 1998). Vesicular acetylcholine transporter (VAChT) and choline acetyltransferase (ChAT) mark cholinergic (presumptively parasympathetic) nerve terminals. The sensory peptidergic markers CGRP, substance P, and TRPV1 label subsets of spinal and vagal afferent fibers (Spencer et al., 2018; Berthoud et al., 2024). Neurofilament 200 (NF200/NFH) identifies myelinated fibers, which in the liver are primarily preganglionic parasympathetic axons.

A critical methodological point is that no single marker unambiguously identifies the source of a nerve fiber. TH is expressed by both sympathetic postganglionic neurons and a small subset of sensory neurons; VAChT immunoreactivity in human and mouse liver has yielded inconsistent results across studies (Weihe et al., 2005; Chen et al., 2024); and CGRP labels both spinal DRG-derived and vagal nodose-derived sensory fibers. The field would benefit from standardized multi-marker panels applied to the same specimen, combined with regional annotation (portal vs. lobular vs. capsular), to enable reliable cross-study comparisons.

Furthermore, antibody performance varies substantially between species and tissue conditions. VAChT antibodies, in particular, have produced discrepant results in mouse liver: some 3D imaging studies report negligible VAChT-positive parenchymal fibers (Adori et al., 2021), while genetic labeling approaches using Cre-dependent reporters expressed from the ChAT locus reveal cholinergic terminals in periportal and midlobular regions (Hwang et al., 2024). These discrepancies likely reflect a combination of low antigen abundance, antibody accessibility in myelinated preganglionic fibers, and the punctate morphology of cholinergic en passant synapses, rather than a true absence of cholinergic innervation.

### 3.2 Neural tracing approaches

Definitive identification of the source, trajectory, and target of hepatic nerve fibers requires neural tracing. Several approaches have been applied:

Classical tracer injection with Fluoro-Gold, cholera toxin B subunit (CTB), or wheat germ agglutinin-horseradish peroxidase (WGA-HRP) into the liver or portal vein region can retrogradely label neurons in the celiac ganglia, nodose ganglia, and DRG, confirming the multi-source origin of hepatic innervation (Strack et al., 1989; Wheatley et al., 1993). These tracers are limited by diffusion away from the injection site and by uptake by fibers of passage.

A complementary strategy uses retrograde adeno-associated virus (AAV) serotypes (e.g., AAVrg, AAV.Php.eB), which can transduce axon terminals after hepatic injection and undergo retrograde transport to the cell body, where Cre-dependent expression of fluorescent reporters enables cell-type-specific labeling. This approach has been used to identify DMV cholinergic neurons projecting to the mouse liver (Hwang et al., 2024) and liver-projecting vagal sensory neurons in the nodose ganglia (Hwang et al., 2025).

Cell-type specificity can be further improved with Cre-dependent genetic tracing in transgenic mice expressing Cre recombinase under cell-type-specific promoters (e.g., ChAT-Cre for cholinergic neurons, Phox2b-Cre for visceral sensory neurons, Th-Cre for catecholaminergic neurons), Cre-dependent AAVs injected into the liver or nodose ganglia enable selective labeling of defined neuronal populations and their peripheral terminals (Hwang et al., 2024; Hwang et al., 2025).

For multisynaptic circuit mapping, pseudorabies virus (PRV) tracers cross synapses in the retrograde direction, enabling identification of multi-synaptic circuits. Injection of PRV into the mouse liver labels DMV neurons within 48 hours and hypothalamic paraventricular nucleus (PVN) neurons within 72 hours, confirming the existence of polysynaptic brain–liver pathways (Stanley et al., 2010).

Functional interrogation can then be achieved with chemogenetic and optogenetic approaches. Expression of designer receptors exclusively activated by designer drugs (DREADDs) or channelrhodopsin in liver-projecting neurons enables selective manipulation of defined neural populations. For example, chemogenetic silencing of liver-projecting vagal sensory neurons prevents diet-induced hepatic steatosis and reduces anxiety-like behavior (Hwang et al., 2025).

Surgical transection of the common hepatic branch of the vagus nerve remains widely used to assess vagal contribution to hepatic function, but this approach carries a major confound: the common hepatic branch also supplies the pylorus, duodenum, and pancreas via the gastroduodenal branch, meaning that metabolic effects of hepatic branch vagotomy cannot be attributed to loss of hepatic innervation alone (Berthoud, 2004; Berthoud et al., 2024).

A pervasive concern across all tracing methods is tracer spread. AAV injected into the liver parenchyma can leak into the systemic circulation, transducing neurons in distant organs; Fluoro-Gold can diffuse into the peritoneal cavity; and PRV can spread transsynaptically to circuits unrelated to the liver if the initial infection is not strictly confined. These confounds are particularly problematic for human translation, where tracing approaches are essentially unavailable.

### 3.3 Tissue clearing and volumetric imaging

The transition from 2D histology to 3D volumetric imaging of hepatic innervation has been enabled by the development of tissue clearing protocols optimized for the liver.

Among tissue-clearing approaches, aqueous-based protocols, including CUBIC (Clear, Unobstructed Brain/Body Imaging Cocktails and computational analysis) and its liver-optimized derivative Liver-CUBIC, achieve delipidation and refractive index matching with minimal tissue shrinkage (Xu et al., 2026). Organic solvent-based methods such as iDISCO and iDISCO+ provide superior transparency and are well-suited to light-sheet microscopy but may quench endogenous fluorescence and cause tissue shrinkage (Renier et al., 2014). Ethyl cinnamate (ECi)-based clearing offers a non-toxic alternative compatible with clinical biopsy workflows (Jacob et al., 2024). More recently, high-refractive-index polymer embedding combined with photochemical bleaching of autofluorescent pigments (lipofuscin, bilirubin, porphyrins) has enabled super-resolution imaging of human liver specimens (Chen et al., 2024; Lee et al., 2025).

For volumetric acquisition, light-sheet fluorescence microscopy (LSFM) is the method of choice for imaging large liver volumes (mm³ to cm³ scale) at cellular resolution with minimal photobleaching (Jacob et al., 2024). Confocal microscopy with Airyscan detection provides sub-diffraction resolution (~120 nm lateral) suitable for resolving individual axonal varicosities and their spatial relationship to target cells (Chen et al., 2024). For ultrastructural analysis, serial block-face scanning electron microscopy (SBF-SEM) can resolve synaptic vesicles and neuro-effector junction morphology, though it is limited to very small volumes.

After volumetric acquisition, computational pipelines perform nerve fiber segmentation (skeletonization), branch-point detection, and spatial co-localization analysis with vascular, biliary, and cellular markers (Adori et al., 2021; Xu et al., 2026). Machine learning-based segmentation approaches, including WEKA and deep learning models, are increasingly used to automate nerve fiber tracing in cleared liver specimens (Jacob et al., 2024).

Applying these methods to human liver introduces challenges not encountered in rodent tissue: intense autofluorescence from lipofuscin, bilirubin, and porphyrins; variable post-mortem or intraoperative ischemic intervals that degrade antigenicity; steatosis-induced light scattering that impairs clearing and imaging depth; and photobleaching during extended acquisition (Lee et al., 2025). A recent comprehensive review identified immediate fixation, protease inhibitor supplementation, and forensic-pathology-based quality control as essential prerequisites for successful human liver neurohistology (Lee et al., 2025).

### 3.4 Integration with spatial and single-cell omics

The structural maps generated by 3D imaging must ultimately be linked to molecular identity—knowing which neuron projects to which liver cell requires knowing what receptors that cell expresses.

At the molecular level, single-cell and single-nucleus RNA sequencing (sc/snRNA-seq) has generated transcriptomic atlases of the mouse and human liver that catalogue receptor expression across hepatocytes, cholangiocytes, HSCs, Kupffer cells, and endothelial cells, revealing that adrenergic, cholinergic, and neuropeptide receptors are expressed in highly cell-type-specific patterns (MacParland et al., 2018; Ramachandran et al., 2019). Complementary transcriptomic profiling of nodose ganglion neurons has identified molecularly defined subtypes of vagal sensory neurons, including a subset that projects to the liver and co-expresses markers of multimodal C-fiber nociceptors (Hwang et al., 2025).

Spatial transcriptomic technologies such as Visium (10x Genomics) and MERFISH can map gene expression onto tissue sections with spatial resolution of approximately 10–100 μm, enabling identification of receptor-expressing cells in defined anatomical zones. These datasets can be used to test whether adrenergic, cholinergic, sensory, and neurotrophin-receptor programs are enriched near mapped nerve fibers, but transcript localization alone does not establish neural connectivity (Guilliams et al., 2022).

Complementary spatial proteomic approaches, including multiplexed immunohistochemistry platforms such as CODEX, MIBI, and CyCIF, can co-register 20–60 protein markers on a single tissue section, enabling simultaneous visualization of nerve fibers, their molecular identity (TH, VAChT, CGRP), and the receptor expression profile of adjacent cells (Goltsev et al., 2018).

At the computational level, tools such as CellPhoneDB, NicheNet, and CellChat can predict ligand–receptor interactions between nerves and local cells based on scRNA-seq data, generating hypotheses about functional connectivity that can then be tested by anatomical proximity analysis (Efremova et al., 2020).

A critical caveat applies to all molecular approaches: the presence of a receptor transcript or protein in a cell near a nerve terminal does not, by itself, constitute evidence of a functional neuro-effector connection. Spatial transcriptomics can identify a hepatocyte expressing β2-adrenoceptor mRNA adjacent to a TH-positive varicosity, but functional validation requires demonstrating that norepinephrine release from that terminal produces a measurable cellular response. Bridging structural and molecular evidence to functional connectivity remains the central unresolved challenge in hepatic neurobiology.

### 3.5 Quantitative standards

To enable reproducible cross-study comparisons, we propose that future studies reporting hepatic neural architecture should include, at minimum, the following quantitative metrics:

- Total nerve fiber volume and length (per unit tissue volume)
- Branch-point density (number per mm³)
- Periportal nerve density (nerve area/portal tract area)
- Nerve-to-vessel distance distribution (mean, median, and histogram)
- Nerve-to-cholangiocyte distance distribution
- Periportal-to-intralobular nerve fiber ratio
- Proportion of nerve fibers showing degenerative morphology (swelling, fragmentation, beading)
- Proportion of nerve fibers showing sprouting morphology (fine caliber, growth cone-like endings)

These metrics should be stratified by nerve subtype (sympathetic, parasympathetic, sensory, peptidergic) where multi-marker data are available, and reported separately for each hepatic zone and lobular region. A comparative overview of available technologies is provided in **Table 1**.

**Table 1:** Comparison of three-dimensional liver neural imaging, tracing, and spatial omics technologies.

Technology	Spatial resolution	Tissue volume	Strengths	Limitations	Key refs
LSFM + CUBIC/Liver-CUBIC	1–5 μm	mm³–cm³	Large volume, minimal photobleaching, multicolor compatible	Resolution insufficient for varicosities; autofluorescence	(Renier et al., 2014; Xu et al., 2026)
LSFM + iDISCO	1–5 μm	mm³–cm³	Superior transparency, established protocol	Tissue shrinkage, fluorescence quenching	(Renier et al., 2014)
LSFM + ECi clearing	1–5 μm	mm³	Non-toxic, cost-effective, clinical biopsy-compatible	Limited human validation	(Jacob et al., 2024)
Confocal + Airyscan + polymer clearing	~120 nm lateral	μm³–mm³	Super-resolution, resolves varicosities, human-compatible	Limited depth, photobleaching	(Chen et al., 2024; Lee et al., 2025)
SBF-SEM	~5 nm (z)	μm³	Ultrastructural resolution, resolves synaptic vesicles	Extremely limited volume, destructive	(Ohata, 1984)
Fluoro-Gold/CTB tracing	Cellular	Whole organ	Established, inexpensive	Tracer diffusion, fibers-of-passage uptake	(Strack et al., 1989; Wheatley et al., 1993)
Retrograde AAV (AAVrg, AAV.Php.eB)	Cellular	Whole organ	Cell-type specificity with Cre, high transduction	Extra-hepatic transduction possible; no human use	(Hwang et al., 2024; Hwang et al., 2025)
PRV transsynaptic tracing	Multi-synaptic	Whole circuit	Maps polysynaptic pathways	Spread beyond target circuit, biosafety	(Stanley et al., 2010)
Genetic tracing (Cre-lox)	Cellular	Whole organism	Definitive cell-type identification	Transgenic models only; not human-applicable	(Hwang et al., 2024; Hwang et al., 2025)
Visium spatial transcriptomics	55 μm spots	Tissue section	Unbiased gene expression, receptor mapping	Resolution limits single-cell assignment; no functional proof	(Guilliams et al., 2022)
MERFISH	~100 nm	Tissue section	Subcellular resolution, hundreds of genes	Specialized equipment, limited protein data	—
Multiplexed IHC (CODEX, CyCIF)	~200 nm	Tissue section	20–60 protein markers simultaneously	Antibody-dependent, panel constraints	(Goltsev et al., 2018)
Ligand–receptor prediction (CellPhoneDB, NicheNet)	Computational	Transcriptome-wide	Hypothesis generation from scRNA-seq	Predictions require anatomical and functional validation	(Efremova et al., 2020)
Proximity analysis (3D nerve–cell distance)	~200 nm	mm³	Direct spatial evidence for neuro–cellular associations	Does not prove functional connectivity	(Adori et al., 2021; Chen et al., 2024; Xu et al., 2026)

LSFM, light-sheet fluorescence microscopy; CUBIC, Clear Unobstructed Brain/Body Imaging Cocktails; ECi, ethyl cinnamate; SBF-SEM, serial block-face scanning electron microscopy; CTB, cholera toxin B subunit; AAV, adeno-associated virus; PRV, pseudorabies virus; IHC, immunohistochemistry.

## 4 Three-dimensional architecture of the hepatic nervous system

### 4.1 Entry routes and major hepatic plexuses

Nerve fibers reach the liver via three principal conduits that travel together within the Glissonian sheath: the hepatic artery, the portal vein, and the bile duct (Akiyoshi et al., 1998; Xu et al., 2026). The anterior hepatic plexus, derived from the celiac plexus and left vagus nerve, enters at the hepatic hilum alongside the hepatic artery. The posterior hepatic plexus, derived from the right celiac plexus and right vagus nerve, accompanies the portal vein. These two plexuses intercommunicate extensively within the porta hepatis and along the major portal tracts, forming a continuous perivascular neural network (Lin et al., 1995; Akiyoshi et al., 1998).

Within the liver, nerve bundles branch in parallel with the branching portal vein and hepatic artery, maintaining close apposition to the vessel wall. At the level of the terminal portal venule, the nerve bundles dissociate into smaller fascicles and individual fibers that either remain in the portal space or enter the hepatic lobule (Xu et al., 2026). The precise point at which fibers leave the portal tract to enter the parenchyma varies by species, nerve subtype, and hepatic zone (Section 7).

Electron microscopy studies in the guinea pig liver have identified two morphologically distinct nerve terminal types in the portal space: adrenergic varicosities containing small granular vesicles that release transmitter into the open portal connective tissue space (without forming classical synaptic contacts), and large, mitochondria-rich presumptive sensory terminals that contact HSCs and hepatocytes (Ohata, 1984). These ultrastructural findings suggest that portal neural signaling occurs predominantly via volume transmission (paracrine neurotransmitter diffusion) rather than via structurally defined synaptic clefts.

### 4.2 Sympathetic innervation

Sympathetic (noradrenergic) innervation constitutes the quantitatively dominant neural input to the mammalian liver. Postganglionic sympathetic neurons in the celiac and superior mesenteric ganglia send TH-positive, NPY-co-expressing axons to the liver via the splanchnic nerves (Strack et al., 1989; Lin et al., 1995).

3D imaging studies consistently demonstrate that sympathetic fibers are concentrated in the portal tract, where they form dense perivascular and periductal plexuses (Adori et al., 2021; Chen et al., 2024; Xu et al., 2026). From the portal tract, TH-positive fibers extend into the hepatic lobule, but the depth of penetration is strongly species-dependent. In the mouse and rat, sympathetic fibers are largely confined to the immediate periportal region (zone 1), with sparse or absent intralobular fibers (Ding et al., 1994; Akiyoshi et al., 1998; Adori et al., 2021). In contrast, the guinea pig, dog, pig, nonhuman primate, and human liver all show TH-positive fibers penetrating into the lobular parenchyma, where varicosities approach hepatocytes and HSCs (Ding et al., 1994; Lin et al., 1995; Akiyoshi et al., 1998).

In human liver, 3D/Airyscan super-resolution imaging has demonstrated that TH-positive sympathetic varicosities extend from the portal space into the hepatic lobule and form close appositions with hepatocytes, particularly ballooning hepatocytes in metabolic dysfunction-associated steatohepatitis (MASH) (Chen et al., 2024). The density of intralobular sympathetic innervation in human liver is approximately 2.6-fold higher than in human pancreas, underscoring that the liver is a richly innervated parenchymal organ—at least in humans (Chen et al., 2024).

Functionally, hepatic sympathetic nerves regulate glucose output (via α1- and β2-adrenoceptors on hepatocytes), hepatic blood flow (via α1-mediated vasoconstriction of the hepatic artery and portal vein), and bile duct secretion (via α1-adrenoceptors on cholangiocytes) (Mizuno and Ueno, 2017; Ehrlich et al., 2018; Borgmann and Fenselau, 2024). The spatially restricted distribution of sympathetic fibers—highest density in the periportal region where gluconeogenic hepatocytes are concentrated—is consistent with the zone-specific metabolic demands placed on the liver.

### 4.3 Parasympathetic innervation and anatomical controversy

Whether the liver receives functionally significant parasympathetic (cholinergic) innervation, and whether cholinergic fibers directly contact hepatocytes, is arguably the most contentious question in hepatic neuroanatomy. We argue that this controversy arises from three conflated issues that must be disentangled, which in turn reflect four distinct forms of evidence that are frequently collapsed: physiological evidence for vagal regulation of hepatic function, anatomical evidence for cholinergic nerve fibers, anatomical evidence for direct nerve–hepatocyte contacts, and functional evidence for neurotransmission at such contacts. These forms of evidence differ substantially in strength and in what each can establish, and keeping them distinct resolves much of the apparent contradiction.

Physiological evidence for vagal regulation should first be distinguished from direct cholinergic innervation of hepatocytes. There is overwhelming physiological evidence that the vagus nerve influences hepatic glucose and lipid metabolism, bile secretion, and inflammation (Borovikova et al., 2000; Mizuno and Ueno, 2017; Borgmann and Fenselau, 2024; Hwang et al., 2024). However, vagal effects on the liver may be mediated through several mechanisms that do not require direct cholinergic synapses onto hepatocytes: (a) cholinergic regulation of pancreatic hormone secretion (insulin, glucagon), which then reaches the liver via the portal vein; (b) cholinergic modulation of hepatic blood flow and sinusoidal perfusion; (c) cholinergic signaling to cholangiocytes and Kupffer cells within the portal tract; (d) vago-vagal reflexes in which vagal afferent signals from the liver trigger vagal efferent output to the pancreas and gastrointestinal tract (Berthoud, 2004; Berthoud et al., 2024). Demonstrating that vagotomy alters hepatic function does not, by itself, establish that the vagus nerve directly innervates hepatocytes.

A second source of uncertainty arises from discrepant VAChT/ChAT immunoreactivity across studies. Some 3D imaging studies in mouse liver report negligible VAChT-positive fibers in the hepatic parenchyma, leading to the conclusion that hepatic innervation is “predominantly sympathetic” (Adori et al., 2021). In contrast, other groups have detected VAChT-positive nerve terminals around bile ducts in human liver (Chen et al., 2024) and, using genetic approaches (ChAT-Cre-driven PLAP expression), have demonstrated cholinergic nerve fibers in periportal and midlobular regions of mouse liver (Hwang et al., 2024). These discrepancies may be explained by technical limitations: parasympathetic preganglionic fibers are thinly myelinated (unlike unmyelinated sympathetic postganglionic fibers), which may reduce antibody accessibility; the en passant varicosities of cholinergic terminals are small and punctate; and VAChT protein abundance in peripheral nerve terminals is substantially lower than in central cholinergic synapses, pushing detection to the limit of immunohistochemical sensitivity (Weihe et al., 2005).

Interpretation is further complicated by non-neuronal sources of acetylcholine. Acetylcholine is not exclusively a neuronal transmitter. Hepatocytes, cholangiocytes, Kupffer cells, and HSCs all express components of the cholinergic system, including ChAT and acetylcholinesterase (Shimazu, 1996; Wessler and Kirkpatrick, 2008). Locally synthesized acetylcholine may act in an autocrine or paracrine manner through muscarinic and nicotinic receptors independently of neural input. Thus, detecting acetylcholine or ChAT in liver tissue does not prove the presence of cholinergic nerve fibers.

Taken together, these findings support a level-of-evidence interpretation that separates four distinct questions. Direct human evidence demonstrates cholinergic innervation of portal and biliary structures: VAChT-positive terminals have been visualized around septal bile ducts in human liver (Chen et al., 2024). Evidence for periportal cholinergic fibers is more limited and derives substantially from animal studies: genetic tracing in mice demonstrates cholinergic projections from the DMV to periportal and midlobular regions (Hwang et al., 2024), but comparable direct demonstration in human tissue is lacking. Direct cholinergic innervation of parenchymal hepatocytes remains uncertain in both species and has not been established by functional criteria in humans. Functional evidence for vagal regulation of hepatic glucose and bile handling is robust in humans (Borgmann and Fenselau, 2024), but this does not localize the effect to cholinergic terminals contacting hepatocytes. The contradictory evidence comprises (i) 3D imaging studies reporting negligible parenchymal VAChT immunoreactivity in mouse liver (Adori et al., 2021); (ii) the relative paucity of human cholinergic data; and (iii) the well-documented existence of non-neuronal acetylcholine sources in the liver (Shimazu, 1996; Wessler and Kirkpatrick, 2008). We therefore interpret the field as follows: cholinergic innervation of the liver is real but concentrated around portal, periductal (biliary), and limited periportal territories, whereas direct cholinergic innervation of hepatocytes—if it occurs—is sparse, species-dependent, and not yet demonstrated by functional criteria. Many vagal effects on hepatic metabolism are likely mediated indirectly, through modulation of pancreatic hormone secretion, hepatic blood flow, and non-neuronal cholinergic signaling. The relative contribution of direct versus indirect vagal regulation likely varies by species, metabolic state, and disease condition, and resolving this question will require combined genetic labeling, 3D imaging, and functional assessment in both rodent models and human tissue.

### 4.4 Sensory afferent architecture

Hepatic sensory innervation originates from two anatomically and functionally distinct populations of primary afferent neurons.

Vagal sensory neurons innervating the liver have cell bodies that reside in the nodose ganglia and project centrally to the NTS. Peripherally, their terminal fields are concentrated in the periportal region, particularly around the portal vein, bile ducts, and in the connective tissue of the portal space (Berthoud and Powley, 1993; Hwang et al., 2025). A 2025 study using retrograde AAV tracing from the mouse liver demonstrated that liver-projecting vagal sensory neurons are a molecularly distinct subpopulation that co-expresses markers of multimodal C-fiber afferents, including TRPV1, and are concentrated in the periportal area (Hwang et al., 2025). These neurons respond to a broad range of stimuli, including glucose, lipids, bile acids, cytokines, osmolarity, and mechanical distension (Section 5.2).

By contrast, spinal sensory neurons in thoracic DRG (T7–T13) send peripheral processes to the liver via the splanchnic nerves, with central processes terminating in laminae I, II, and V of the spinal dorsal horn (Spencer et al., 2018; Berthoud et al., 2024). These fibers are predominantly peptidergic (CGRP-positive, substance P-positive, TRPV1-expressing) and are most dense around the bile ducts, portal vein, and in the hepatic capsule (Glisson’s capsule) (Ding et al., 1994; Akiyoshi et al., 1998). Capsular sensory innervation is particularly rich and is thought to mediate the sensation of liver capsular distension that produces the characteristic right upper quadrant pain associated with hepatic congestion and inflammation.

The distribution of sensory terminals also differs across hepatic structures. The portal vein wall contains a dense plexus of sensory fibers, particularly in the adventitia, where they are positioned to detect changes in portal pressure and blood composition (Berthoud et al., 2024). The bile ducts receive both vagal and spinal sensory innervation, concentrated in the periductal connective tissue; this innervation is remodeled during cholestatic injury (Section 6.3). The hepatic parenchyma contains fewer sensory fibers than the portal tract, but CGRP- and TRPV1-positive fibers have been identified in the space of Disse in some species, in proximity to HSCs and hepatocytes (Ding et al., 1994; Akiyoshi et al., 1998).

### 4.5 The periportal lamellar complex as a candidate neural scaffold

A 2026 study combining multicolor metallic compound nanoparticle perfusion with an optimized Liver-CUBIC clearing protocol identified a previously unrecognized anatomical structure in the mouse liver: the periportal lamellar complex (PLC) (Xu et al., 2026). The PLC is a CD34^+^Sca-1^+^CD31^+^ endothelial sheet that encircles the portal vein in its outer (adventitial) layer, forming multi-branched extensions that radiate from the main portal vein into the surrounding tissue.

Three features of the PLC make it particularly relevant to hepatic neural organization (Mizuno and Ueno, 2017): Spatial association with sympathetic nerves—TH-positive sympathetic fibers are arranged in a characteristic “beads-on-a-string” pattern around and along the PLC, suggesting that this endothelial structure provides a physical scaffold for sympathetic fiber distribution along the portal vein (Xu et al., 2026) (Borgmann and Fenselau, 2024). Molecular signature consistent with axonal guidance—single-cell transcriptomic analysis of the CD34^+^Sca-1^+^ endothelial population that forms the PLC revealed upregulation of neurodevelopmental and axonal guidance genes, including semaphorins, ephrins, and netrin-family members, suggesting that the PLC actively patterns neural growth rather than merely providing passive support (Xu et al., 2026) (Berthoud and Neuhuber, 2000). Structural remodeling in fibrosis—in carbon tetrachloride (CCl^4^)-induced liver fibrosis, the PLC extends from the portal vein toward the hepatic lobule, forming structural extensions that appear to guide both bile duct and nerve fiber migration into the expanding fibrotic septum. This observation suggests that the PLC may function as a pathfinding scaffold during pathological tissue remodeling (Xu et al., 2026).

The PLC shares morphological and molecular features with telocytes—CD34-expressing interstitial cells with long, slender processes (telopodes) that have been previously identified in the liver and other organs (Liu et al., 2016). Whether the PLC is a specialized vascular derivative of the telocyte network, or a distinct endothelial structure, remains to be determined.

The PLC discovery raises several important questions: Does a homologous structure exist in the human liver? Is PLC-guided nerve migration during fibrosis a beneficial repair response or a maladaptive process that exacerbates fibrosis (Section 6.4)? Can PLC-associated molecular signals be targeted to promote organized neural regeneration while limiting pathological nerve sprouting? Answers to these questions will require validation of the PLC in human liver tissue and functional studies in genetic models that selectively disrupt PLC-associated guidance cues.

## 5 The periportal region as a dominant interoceptive integration site

### 5.1 Why the periportal niche is a dominant interoceptive integration site

Interoception—the sensing and central transmission of the body’s internal physiological state—is not uniformly distributed across the liver. Anatomical, physiological, and molecular evidence converges on the periportal region as a dominant anatomical integration site for interoception (Berthoud et al., 2024; Hwang et al., 2025), while the hepatic capsule, sinusoidal regions, and metabolic zones also contribute distinct interoceptive signals.

The periportal niche possesses five properties that make it particularly well suited for interoceptive function. First, it is the first hepatic compartment to be exposed to intestinally absorbed nutrients, hormones, and microbial products arriving via the portal vein, enabling early detection of changes in nutrient status, osmolarity, and gut-derived inflammatory signals (Berthoud et al., 2024). Second, it receives pancreatic hormones (insulin, glucagon) and gut peptides (CCK, GLP-1, PYY) at their highest post-secretory concentrations, providing a privileged site for sensing the endocrine milieu. Third, the portal vein wall is densely innervated by mechanosensitive sensory fibers that detect changes in portal pressure and blood flow (Niijima, 1983; Berthoud et al., 2024). Fourth, the periportal space is enriched in resident immune cells, particularly Kupffer cells and dendritic cells, which generate cytokine and chemokine signals that are detected by adjacent sensory nerve terminals (Watkins et al., 1995; Tracey, 2002). Fifth, bile ducts within the portal tract are a source of biliary constituents (bile acids, bicarbonate, secreted proteins) that can be sensed by periductal nerve fibers (Gigliozzi et al., 2004; Ehrlich et al., 2018).

This multi-modal convergence of nutrient, hormonal, mechanical, microbial, and inflammatory signals within a spatially restricted, densely innervated anatomical compartment characterizes the periportal region as a dominant interoceptive integration site, although the capsule and sinusoidal compartments carry additional, partially non-overlapping sensory modalities.

### 5.2 Molecular sensors of hepatic afferent neurons

Hepatic sensory neurons express a diverse array of molecular sensors that transduce specific physical and chemical stimuli into neural signals. For each sensor we distinguish, where possible, among four evidentiary levels: demonstrated expression in liver-projecting neurons (Hwang et al., 2025), expression in visceral or somatic sensory neurons generally, inferred hepatic relevance, and speculative physiological significance.

Mechanosensation represents one major component of hepatic afferent signaling. Piezo1 and Piezo2 are mechanically gated cation channels identified as core components of distinct mechanically activated currents (Coste et al., 2010). Among hepatic afferents, Piezo2 expression has been directly demonstrated in liver-projecting vagal sensory neurons by single-nucleus profiling (Hwang et al., 2025). Whether Piezo1—or Piezo2 in spinal afferents—is expressed by neurons that specifically innervate the portal vein and hepatic vasculature has not been directly mapped; their proposed detection of portal pressure and vascular wall stretch is therefore inferred from their established mechanosensitivity and from the presence of mechanosensitive afferent terminals in the portal vein wall (Berthoud et al., 2024).

Chemical and inflammatory cues may also be detected through TRP channels. TRPV1, the capsaicin receptor, is expressed by liver-projecting vagal sensory neurons (Hwang et al., 2025) and is activated by noxious heat (>43 °C), low pH, and lipid metabolites including anandamide and oxidized linoleic acid metabolites (Caterina et al., 1997). TRPA1 is also expressed by liver-projecting vagal sensory neurons (Hwang et al., 2025) and is activated by reactive oxygen species, lipid peroxidation products (4-hydroxynonenal, 4-HNE), and bacterial products, positioning it as a candidate sensor of oxidative stress and gut-derived inflammatory signals in the portal microenvironment; this hepatic role is inferred from its expression profile and general sensory-neuron biology rather than from functional demonstration in liver-projecting afferents. TRPM3 is activated by pregnenolone sulfate, other neurosteroids, and heat, but its expression by hepatic sensory afferents has not been directly demonstrated and remains speculative.

Hepatic afferents may additionally respond to hormonal and nutrient-related signals; for example, the cholecystokinin A receptor (CCKAR) is expressed by vagal sensory neurons and has been implicated in meal-related satiety signaling from the portal region (Berthoud et al., 2024). GLP-1 receptors on vagal afferents have been proposed to contribute to portal glucose sensing and insulin secretion (Mizuno and Ueno, 2017), although direct demonstration on liver-projecting neurons is limited. SGLT3, a sodium–glucose co-transporter that functions as a glucose sensor rather than a transporter, is expressed in the portal region and mediates glucose-induced neuronal activation through a vagal-independent, spinal afferent pathway (Delaere et al., 2012). Neuropeptide Y receptor type 2 (NPY2R) expression on periportal sympathetic and sensory fibers has been suggested but not directly mapped in the liver.

Inflammatory sensing is supported more cautiously by evidence for cytokine and mediator receptors. Hepatic sensory neurons are proposed to express receptors for tumor necrosis factor (TNFR1), interleukin-1β (IL-1R), and interleukin-6 (IL-6R/gp130), enabling detection of hepatic inflammation; however, direct demonstration of these receptors on liver-projecting neurons is limited, and much of the supporting evidence derives from vagal afferents generally rather than from the liver specifically (Watkins et al., 1995). P2X purinergic receptors (particularly P2X2 and P2X3) detect ATP released from stressed or damaged cells, but their expression on hepatic afferents has not been directly mapped. The 5-HT3 serotonin receptor is expressed by vagal afferents generally and may mediate detection of serotonin released from enterochromaffin cells and platelets in the portal circulation; this hepatic role remains speculative.

A 2025 study in Nature Communications provided the most comprehensive molecular characterization to date of liver-projecting vagal sensory neurons, demonstrating that these neurons exhibit multimodal C-fiber properties, co-express TRPV1 and markers of nociceptive afferents, and are concentrated in the periportal region, where they detect signals that drive both metabolic adaptations (hepatic steatosis, insulin resistance) and anxiety-related behavior in diet-induced obese mice (Hwang et al., 2025).

### 5.3 Local neurovascular and neuroimmune interactions

Within the periportal niche, nerve fibers engage in spatially defined interactions with specific non-neural cell types.

At the nerve–cholangiocyte interface, cholangiocytes express M3 muscarinic receptors that, upon acetylcholine binding, potentiate secretin-stimulated bicarbonate secretion via a calcineurin-dependent cross-talk mechanism between Ca²^+^ and cAMP signaling (Alvaro et al., 1997). Sympathetic fibers release norepinephrine that acts on α1-adrenoceptors to stimulate cholangiocyte proliferation and on β-adrenoceptors to modulate ductal secretion (Ehrlich et al., 2018). In cholestasis, cholangiocytes upregulate NGF secretion, which stimulates sensory and sympathetic nerve fiber growth in the periductal space, creating a positive-feedback loop between biliary injury and neural remodeling (Gigliozzi et al., 2004).

Around the portal vein endothelium, sympathetic varicosities in the portal vein adventitia release norepinephrine, which diffuses to vascular smooth muscle and endothelial cells to regulate portal pressure and flow. Sensory terminals in the vein wall detect stretch, osmolarity, and glucose concentration, transmitting this information centrally via vagal and spinal afferent pathways (Niijima, 1983; Berthoud et al., 2024).

Neuroimmune signaling involving Kupffer cells is also plausible because Kupffer cells express adrenergic and cholinergic receptors, providing a plausible substrate for neuroimmune modulation. Systemic vagus nerve stimulation suppresses inflammatory responses in experimental models (Borovikova et al., 2000; Tracey, 2002), whereas Kupffer-cell-derived TNFα contributes to sympathetic neuropathy in high-fat-fed mice (Adori et al., 2021). However, direct liver-specific neural control of Kupffer cells has not been anatomically and functionally demonstrated in humans.

Interactions with HSCs provide another potential neurofibrogenic interface because HSCs are positioned near sinusoidal and periportal neural elements and respond to cholinergic and adrenergic cues. Acetylcholine promotes proliferation and collagen gene expression in myofibroblastic HSCs (Oben et al., 2003), while p75NTR links neurotrophin signaling to HSC fate (Passino et al., 2007). Pharmacological studies also suggest that adrenergic modulation can influence fibrogenic activity, although the cellular and liver-specific mechanisms require further validation.

For hepatocytes, direct neural apposition is species dependent. In species where sympathetic fibers extend into the lobule, TH-positive varicosities approach hepatocytes within the space of Disse. Norepinephrine released from these varicosities acts on hepatocyte α1- and β2-adrenoceptors to stimulate glycogenolysis and gluconeogenesis (α1, β2) and inhibit glycogen synthesis (β2). Whether this signaling occurs via structured neuro-effector junctions or via paracrine volume transmission remains unclear; electron microscopy studies in multiple species have identified both close appositions (20–100 nm gap) between nerve varicosities and hepatocytes and, more commonly, bare varicosities releasing transmitter into the interstitial space (Ohata, 1984).

The extracellular matrix also shapes local neural organization because the basement membrane and interstitial collagen of the portal tract and space of Disse provide the physical substrate along which nerve fibers extend. In fibrosis, excessive collagen deposition physically displaces nerve terminals from their targets and may present a mechanical barrier to regeneration (Liu et al., 2021; Mizuno et al., 2021). Conversely, nerve-derived signals (norepinephrine, NPY) can stimulate collagen production by HSCs and portal fibroblasts, contributing to matrix remodeling (Oben et al., 2003).

### 5.4 Ascending liver-to-brain pathways

Hepatic interoceptive signals ascend to the brain via two anatomically and functionally distinct pathways.

Along the vagal afferent pathway, metabolic, osmotic, and hormonal signals detected by vagal sensory terminals in the periportal space are transmitted via the hepatic branch of the vagus nerve to pseudounipolar neuron cell bodies in the nodose ganglia, and from there to the NTS and area postrema in the dorsal medulla (Berthoud and Powley, 1993; Berthoud et al., 2024). From the NTS, hepatic interoceptive signals are relayed to the parabrachial nucleus, hypothalamus (PVN, lateral hypothalamus, arcuate nucleus), amygdala, and insular cortex, where they influence food intake, energy expenditure, glucose homeostasis, and affective state (Li, 2023; Hwang et al., 2025). This vagal afferent pathway is primarily responsible for conveying information about physiological (non-noxious) hepatic states and is essential for the normal regulation of energy balance and glucose metabolism.

Along the spinal afferent pathway, noxious, inflammatory, and mechanical signals detected by spinal sensory terminals in the liver are transmitted via the splanchnic nerves to DRG cell bodies (T7–T13) and then to the spinal dorsal horn (Spencer et al., 2018; Berthoud et al., 2024). From the spinal cord, hepatic nociceptive signals ascend via the spinothalamic and spinoreticular tracts to the thalamus, hypothalamus, and brainstem, contributing to the perception of hepatic pain (e.g., capsular distension) and to reflex sympathetic activation in response to hepatic inflammation.

These two pathways are not strictly segregated: some hepatic sensory neurons project via both vagal and spinal routes, and there is extensive convergence of vagal and spinal afferent input onto common brainstem targets, particularly the NTS and parabrachial nucleus (Berthoud et al., 2024).

### 5.5 Physiological outputs

The physiological processes regulated by hepatic neural signaling, mediated through the pathways described above, include:

One major physiological output is hepatic glucose regulation. Experimental evidence, derived largely from rodent studies, indicates that sympathetic activation stimulates glycogenolysis and gluconeogenesis while inhibiting glycogen synthesis, increasing hepatic glucose release during fasting, stress, and hypoglycemia. Vagal afferent signaling from portal glucose sensors modulates the counter-regulatory response to hypoglycemia (Mizuno and Ueno, 2017; Li, 2023); whether these portal glucose-sensing mechanisms operate equivalently in humans remains incompletely defined.

Hepatic neural signaling also influences lipid metabolism. Sympathetic innervation promotes hepatic VLDL-triglyceride secretion and fatty acid uptake. In mice, liver-projecting vagal sensory neurons and liver-innervating parasympathetic cholinergic (efferent) neurons are both required for the development of diet-induced hepatic steatosis, such that their loss limits hepatic lipid accumulation (Hwang et al., 2024; Hwang et al., 2025). This identifies vagal afferent and cholinergic efferent pathways as promoters—not suppressors—of steatosis in this context, and cautions against a single generalized claim of “vagal protection” against steatosis.

Beyond liver-intrinsic metabolism, hepatic afferent signaling contributes to food intake and energy expenditure. Portal vein glucose and lipid sensors signal via vagal afferents to the NTS and hypothalamus to promote satiety and increase energy expenditure. Loss of liver-projecting vagal sensory neurons in mice prevents diet-induced obesity—in association with increased energy expenditure—and limits the progression of hepatic steatosis (Hwang et al., 2025), indicating that these afferents promote both weight gain and hepatic lipid accumulation under obesogenic conditions.

Neural control also extends to bile secretion. In experimental models, vagal cholinergic signaling stimulates cholangiocyte bicarbonate secretion (via M3 receptors) and gallbladder contraction, facilitating postprandial bile delivery to the intestine (Alvaro et al., 1997; Ehrlich et al., 2018).

With respect to inflammation, the systemic inflammatory reflex—in which vagus nerve stimulation attenuates pro-inflammatory cytokine responses—is well established in experimental models (Borovikova et al., 2000; Tracey, 2002). However, direct hepatic vagal regulation of Kupffer cells has not been anatomically and functionally established, particularly in humans; apparent cholinergic anti-inflammatory effects in the liver could reflect systemic vagal actions, indirect modulation of splenic or circulating immune cells, and non-neuronal cholinergic signaling rather than direct nerve–Kupffer cell neurotransmission. Sympathetic signaling can promote or suppress inflammation depending on adrenergic receptor subtype and context.

Hepatic interoceptive signaling may also influence anxiety- and stress-related behavior. Liver-projecting vagal sensory neurons influence anxiety-like behavior in mice; selective ablation of these neurons reduces anxiety in diet-induced obese animals, suggesting that hepatic interoceptive signals contribute to affective state (Hwang et al., 2025).

## 6 Remodeling of the hepatic neural niche in disease

This section focuses specifically on how the spatial structure of the hepatic neural niche changes during chronic liver disease, rather than providing a general review of neural involvement in liver pathology. A comparative evidence summary is provided in **Table 2**.

**Table 2:** Neural structural changes in human liver disease: evidence and confidence levels.

Disease	Structural change	Evidence source	Human evidence	Evidence level	Key refs
MASLD/Simple Steatosis	Mild sympathetic axonal swelling, beading, sprouting	Mouse HFD models; limited human 3D imaging	Partial (small series)	Moderate	(Adori et al., 2021; Liu et al., 2021)
MASH	Sympathetic axonal arbor collapse; nerve degeneration correlates with disease severity	Mouse HFD/MCD models; human 3D/Airyscan	Yes (correlated with NAS)	Moderate–Strong	(Adori et al., 2021; Liu et al., 2021; Chen et al., 2024)
Chronic hepatitis (viral)	Reduced total and sympathetic NFAR in portal tract	Human biopsy IHC (n = 85)	Yes	Strong	(Mizuno et al., 2021)
Advanced fibrosis (F3–F4)	Marked reduction in portal tract NFAR; collagen physical isolation of nerves	Human biopsy IHC (n = 85); mouse CCl^4^ fibrosis 3D imaging	Yes	Strong	(Mizuno et al., 2021; Xu et al., 2026)
Cirrhosis	Nerve fibers in septa; absent in regenerative nodules	Human biopsy IHC	Yes	Strong	(Miyazawa et al., 1988; Mizuno et al., 2021)
Fibrosis regression (post-HCV SVR)	Partial recovery of portal tract NFAR	Human paired biopsy IHC (n = 3)	Yes (limited sample)	Weak–Moderate	(Mizuno et al., 2021)
Cholestasis (BDL, PSC, PBC)	NGF upregulation; periductal neural remodeling	Mouse BDL; human PSC biopsy (NGF expression)	Partial (expression only; no 3D nerve imaging)	Weak	(Gigliozzi et al., 2004; Wu et al., 2016)
Liver transplantation (denervated)	No sympathetic reinnervation ≤30 months; partial perivascular regeneration after 15+ years	Human biopsy catecholamine assay; SYP IHC	Yes	Strong	(Kjaer et al., 1994; Hanabata et al., 2026)
MASLD → MASH progression	Immune cell (TNFα)-driven sympathetic neuropathy drives metabolic worsening	Mouse genetic models (Sarm1 KO, Vav3 KO)	Not tested in human	Strong in mouse; absent in human	(Adori et al., 2021; Liu et al., 2021)
Fibrosis (HSC–nerve crosstalk)	Norepinephrine promotes HSC activation; HSCs produce norepinephrine	Mouse/rat HSC culture; human HSC adrenoceptor expression	Partial	Moderate	(Oben et al., 2003; Medina Pizaño et al., 2023)
PSC	Secretin/let-7a/NGF pathway activation	Mouse BDL and Mdr2 KO; human PSC biopsies	Partial	Moderate	(Wu et al., 2016)

NAS, NAFLD Activity Score; NFAR, nerve fiber areal ratio; BDL, bile duct ligation; SVR, sustained virological response; SYP, synaptophysin; HFD, high-fat diet; MCD, methionine-choline-deficient diet.

### 6.1 MASLD and MASH

Metabolic dysfunction-associated steatotic liver disease (MASLD) and its progressive inflammatory form, MASH, are the most extensively studied conditions with respect to hepatic neural remodeling, owing to the development of 3D imaging tools in rodent models of diet-induced obesity.

A hallmark structural change in MASLD/MASH is progressive degeneration of sympathetic nerve fibers. In both mouse models of diet-induced obesity and human fatty liver specimens, 3D imaging reveals a stereotyped sequence: early-stage disease shows mild axonal swelling and beading, accompanied by aberrant sprouting of fine-caliber fibers; advanced steatohepatitis is characterized by near-complete collapse of the sympathetic axonal arbor in the hepatic parenchyma, with only truncated, fragmented fiber remnants remaining around the portal vein (Adori et al., 2021). The severity of sympathetic nerve loss correlates with histological disease severity in human MASLD (Adori et al., 2021).

Mechanistically, mouse studies implicate both inflammatory injury and chronic sympathetic hyperexcitation in this neuropathy. First, TNFα released from CD11b^+^ F4/80^+^ hepatic immune cells under high-fat diet conditions directly damages sympathetic nerve terminals; genetic deletion of Sarm1, a pro-degenerative NADase activated by axonal injury, protects against diet-induced sympathetic neuropathy (Liu et al., 2021). Second, chronic sympathetic hyperexcitation—likely driven by obesity-associated systemic sympathetic activation—itself drives axonal degeneration, as demonstrated in Vav3-deficient mice, where reduced sympathetic tone protects against nerve loss (Adori et al., 2021).

These observations support a possible metabolic injury–neural degeneration–metabolic worsening loop in which loss of sympathetic innervation is not merely a consequence of metabolic injury but may actively worsen hepatic metabolism. In mice, norepinephrine normally suppresses inflammatory cytokine production by hepatic immune cells and maintains hepatocyte insulin sensitivity; loss of sympathetic neurotransmitter supply removes this protective brake, allowing unchecked immune activation and insulin resistance that further drive steatosis and inflammation (Adori et al., 2021; Liu et al., 2021). Whether this loop operates similarly in humans remains unknown. This creates a self-amplifying cycle in which metabolic injury causes nerve loss, which removes metabolic regulatory control, which worsens metabolic injury.

### 6.2 Fibrosis and cirrhosis

In fibrosis and cirrhosis, a prominent structural consequence of matrix accumulation is physical displacement and isolation of nerve fibers. In advanced fibrosis (METAVIR F3–F4), collagen bundles physically separate nerve fibers from their cellular targets—hepatocytes, cholangiocytes, and vascular smooth muscle—creating a mechanical barrier to neurotransmitter diffusion and structurally disrupting the neural niche (Liu et al., 2021; Mizuno et al., 2021).

A 2021 study of 85 human liver biopsies quantified the nerve fiber areal ratio (NFAR, nerve area divided by portal tract area) using PGP9.5 and TH immunohistochemistry and found that NFAR was significantly reduced in chronic hepatitis and advanced fibrosis compared to normal liver, independent of inflammatory activity grade (Mizuno et al., 2021). Sympathetic (TH-positive) NFAR showed a particularly steep decline from F1 to F4 (p = 0.02), consistent with the preferential vulnerability of sympathetic fibers to fibrotic injury (Mizuno et al., 2021). An earlier study similarly observed that in cirrhosis, nerve fibers increased in fibrotic septa but decreased or disappeared within regenerative nodules (Miyazawa et al., 1988).

Fibrogenic remodeling may also be influenced by neural–HSC signaling, with experimental evidence supporting neural regulation of fibrogenic HSC behavior, but the transmitter- and species-specific mechanisms remain incompletely resolved. Acetylcholine promotes proliferation and collagen gene expression in myofibroblastic HSCs (Oben et al., 2003), and pharmacological studies suggest that adrenergic blockade can attenuate fibrogenic activity (Medina Pizaño et al., 2023). Whether activated HSCs sustain a local catecholamine feedback loop in human liver has not been established.

A further spatial mechanism involves PLC expansion and neural–biliary migration. In the CCl^4^ mouse fibrosis model, the PLC extends from the portal vein adventitia into the developing fibrotic septum, appearing to provide a scaffold along which bile ductules and sympathetic nerve fibers migrate (Xu et al., 2026). It is currently unknown whether this PLC-guided migration represents an organized repair response (directing nerves toward regenerating parenchyma) or a maladaptive process (routing nerves into fibrotic septa where they may further stimulate collagen deposition).

Importantly, available human data suggest that nerve loss may be at least partly reversible after fibrosis regression. In a small series of paired liver biopsies from patients who achieved sustained virological response (SVR) after HCV antiviral therapy, NFAR recovered in parallel with fibrosis regression, suggesting that at least partial sympathetic reinnervation is possible when the fibrotic barrier is removed (Mizuno et al., 2021). This observation is consistent with the known capacity of peripheral sympathetic fibers for collateral sprouting and regeneration when the endoneurial environment is permissive.

### 6.3 Cholestatic and biliary disease

Cholestatic liver diseases—including primary biliary cholangitis (PBC), primary sclerosing cholangitis (PSC), and extrahepatic biliary obstruction—produce a distinct pattern of neural niche remodeling centered on the bile duct.

In cholestatic disease, neural remodeling is concentrated around the periductal compartment. The periductal connective tissue is normally innervated by sympathetic, vagal, and sensory (CGRP/substance P-positive) fibers that regulate cholangiocyte secretion and proliferation (Ehrlich et al., 2018). In cholestasis, cholangiocytes undergo a “ductular reaction”—proliferation of reactive bile ductules accompanied by acquisition of a neuroendocrine phenotype characterized by secretion of NGF, serotonin, and other neuroactive molecules (Gigliozzi et al., 2004; Wu et al., 2016). NGF, acting through its high-affinity receptor TrkA on cholangiocytes and its low-affinity receptor p75^NTR on periductal nerves and HSCs, stimulates both cholangiocyte proliferation and periductal nerve fiber growth (Gigliozzi et al., 2004).

One proposed mechanism in PSC involves the secretin–NGF axis. In PSC, the secretin/secretin receptor axis is upregulated in proliferating cholangiocytes. Secretin signaling suppresses microRNA let-7a, which normally restrains NGF expression; the resulting increase in NGF drives both biliary proliferation and TGF-β1-mediated periductal fibrosis (Wu et al., 2016). Human PSC liver biopsies show elevated secretin, secretin receptor, NGF, and TGF-β1 expression with reduced let-7a compared to healthy controls (Wu et al., 2016).

Important evidence gaps nevertheless remain. Compared to MASLD/MASH, the spatial organization of neural remodeling in cholestatic disease is poorly characterized. No 3D imaging study has systematically mapped nerve fiber changes in PSC or PBC liver tissue, and it is unknown whether nerve loss, aberrant sprouting, or both occur in the periductal niche. The role of sensory innervation in cholestasis-associated pruritus—a major clinical symptom—is supported by indirect evidence (opioid and serotonin systems are implicated) but direct anatomical demonstration of altered sensory fiber density or distribution in cholestatic human liver is lacking.

### 6.4 Sex differences in hepatic neural remodeling

Sex is an underappreciated determinant of hepatic neural organization and its disease-associated remodeling, but the available evidence operates at two distinct levels. At the systemic autonomic level, experimental and human studies indicate that estradiol can alter baroreflex control and autonomic balance, including enhanced vagal activity or sympathetic baroreflex regulation under specific experimental conditions (Saleh and Connell, 2000; Hunt et al., 2001; Caeiro et al., 2023). These observations support hormone-sensitive autonomic regulation but should not be interpreted as direct evidence that estrogen changes hepatic nerve density or liver-specific neurotransmission. At the structural hepatic level, evidence is substantially weaker: whether high-fat-diet-induced sympathetic degeneration, or the TNFα- and Sarm1-dependent mechanisms that contribute to it, differs systematically between male and female animals has not been established (Adori et al., 2021; Liu et al., 2021). Sex-related differences in MASLD prevalence and the acceleration of disease after menopause therefore make hormonal modulation of the hepatic neural niche a plausible hypothesis, but a direct mechanistic link remains unproven. Future studies should incorporate sex-stratified analyses of nerve density, nerve-to-cell proximity, and remodeling and should distinguish systemic autonomic effects from liver-specific structural neural changes.

### 6.5 Reversibility versus maladaptive remodeling

The structural changes described above raise three interrelated questions with direct clinical implications.

Is nerve loss a driver of disease or a consequence of fibrosis? The correlation between nerve fiber loss and fibrosis stage is well-established (Adori et al., 2021; Liu et al., 2021; Mizuno et al., 2021), but the direction of causality is not. In MASLD, sympathetic degeneration precedes significant fibrosis in some models, suggesting that neuropathy may be an early, potentially causal event (Liu et al., 2021). In advanced cirrhosis, however, nerve loss is likely a secondary consequence of collagen deposition and architectural distortion. Longitudinal studies with temporal resolution—possible in rodent models but difficult in humans—are needed to establish the sequence.

Is nerve sprouting a repair response or a driver of pathology? The aberrant axonal sprouting observed in early MASLD (Adori et al., 2021) and the PLC-guided nerve migration in fibrosis (Xu et al., 2026) could represent either an adaptive attempt to restore innervation or a maladaptive process that exacerbates disease. In other organs, sympathetic sprouting after injury can promote arrhythmias (heart), pain (DRG), and inflammation (skin). Whether hepatic nerve sprouting contributes to portal hypertension, metabolic dysregulation, or fibrogenesis is unknown.

Does structural recovery of nerves imply functional recovery? Even where NFAR recovers after fibrosis regression (Mizuno et al., 2021), it is not known whether the regenerated nerves functionally re-innervate their appropriate targets (hepatocytes, cholangiocytes, vascular smooth muscle) with normal neurotransmitter release and receptor coupling. Functional recovery after structural regeneration cannot be assumed and must be experimentally demonstrated.

## 7 Why rodent findings cannot be directly extrapolated to humans

This section provides a systematic analysis of the barriers to cross-species translation in hepatic neurobiology. We argue that these barriers are not merely quantitative (e.g., the human liver is larger) but qualitative, reflecting fundamental differences in innervation architecture, methodology, and the nature of available evidence.

### 7.1 Species-specific innervation patterns

Comparative anatomical studies using standardized immunohistochemical markers across multiple mammalian species have revealed consistent, systematic differences in hepatic innervation between rodents and humans (Ding et al., 1994; Lin et al., 1995; Akiyoshi et al., 1998) (**Table 3**).

**Table 3:** Comparison of hepatic innervation features across mouse, rat, guinea pig, dog, pig, nonhuman primate, and human.

Feature	Mouse	Rat	Guinea pig	Dog	Pig	NHP	Human	Key references
Portal tract nerve density	High (PGP9.5^+^, TH^+^)	High (PGP9.5^+^, TH^+^)	High (PGP9.5^+^, TH^+^)	High (PGP9.5^+^, TH^+^)	High (PGP9.5^+^, TH^+^)	High (PGP9.5^+^, TH^+^)	High (PGP9.5^+^, TH^+^)	(Ding et al., 1994; Lin et al., 1995; Akiyoshi et al., 1998)
Intralobular sympathetic (TH^+^) fibers	Sparse, zone 1 only	Sparse, zone 1 only	Penetrate into lobule	Penetrate into lobule	Penetrate into lobule	Penetrate into lobule	Zones 1–2; fibers contact hepatocytes	(Ding et al., 1994; Lin et al., 1995; Akiyoshi et al., 1998; Adori et al., 2021; Chen et al., 2024)
Direct hepatocyte–nerve contacts	Rare or absent (EM)	Rare or absent (EM)	Present (EM)	Present (EM)	Present (EM)	Present (EM)	Present (EM, 3D/Airyscan)	(Ohata, 1984; Akiyoshi et al., 1998; Chen et al., 2024)
Parasympathetic (VAChT^+^) parenchymal fibers	Controversial (IHC weak; genetic tracing positive)	Limited evidence	Not systematically characterized	Not systematically characterized	Not systematically characterized	Not systematically characterized	Present (bile duct, septal)	(Weihe et al., 2005; Adori et al., 2021; Chen et al., 2024; Hwang et al., 2024)
Sensory (CGRP^+^/SP^+^) fibers	Periportal, periductal, capsular	Periportal, periductal, capsular	Periportal, periductal, capsular	Periportal, periductal, capsular	Periportal, periductal, capsular	Periportal, periductal, capsular	Periportal, periductal, capsular, some intralobular	(Ding et al., 1994; Akiyoshi et al., 1998; Spencer et al., 2018; Berthoud et al., 2024)
Intrahepatic ganglia	Rare/absent	Rare/absent	Rare/absent	Rare/absent	Rare/absent	Rare/absent	Reported in portal hilum	(McCuskey, 2004)
Periportal lamellar complex (PLC)	Present	Not examined	Not examined	Not examined	Not examined	Not examined	Not examined	(Xu et al., 2026)
Tissue accessibility for experiments	High	High	Limited	Limited	Limited	Limited	Very limited	(Chen et al., 2024; Lee et al., 2025)
Functional tracing possible	Yes	Yes (limited)	Limited	Limited	Limited	Limited	No	(Hwang et al., 2024; Hwang et al., 2025)
Dynamic neural activity recording	Possible	Possible	Limited	Limited	Limited	Limited	Not possible	—

EM, electron microscopy; IHC, immunohistochemistry; SP, substance P; NHP, nonhuman primate.

Portal tract innervation is robust and readily demonstrable in both rodents and humans, with dense perivascular and periductal sympathetic and sensory plexuses (Lin et al., 1995; Akiyoshi et al., 1998).

Intralobular innervation differs substantially. In the mouse and rat, TH-positive sympathetic fibers are largely confined to the immediate periportal zone (zone 1) and are sparse or absent in zones 2 and 3 (Ding et al., 1994; Lin et al., 1995; Akiyoshi et al., 1998; Adori et al., 2021). In the human liver, TH-positive fibers penetrate into the lobule, and individual varicosities can be found in direct apposition to hepatocytes, particularly zone 1 and zone 2 hepatocytes (Chen et al., 2024). This difference has been confirmed across multiple studies using both conventional immunohistochemistry and 3D super-resolution imaging (Lin et al., 1995; Akiyoshi et al., 1998; Chen et al., 2024).

Hepatocyte–nerve appositions are correspondingly species-dependent. In guinea pig, dog, pig, nonhuman primate, and human liver, electron microscopy and high-resolution light microscopy have demonstrated close appositions (20–100 nm) between nerve varicosities and hepatocyte membranes (Ohata, 1984; Akiyoshi et al., 1998). In mouse and rat liver, such contacts are rare or absent, and neural signaling to hepatocytes likely occurs predominantly through paracrine volume transmission from periportal nerve terminals (Akiyoshi et al., 1998; Adori et al., 2021).

Parasympathetic innervation is where species differences are most consequential. Human liver shows VAChT-positive cholinergic terminals around septal bile ducts (Chen et al., 2024), and functional evidence for vagal regulation of human hepatic glucose metabolism is robust (Borgmann and Fenselau, 2024). In mouse liver, VAChT immunoreactivity in the parenchyma is weak or absent in 3D imaging studies (Adori et al., 2021), yet genetic tracing reveals cholinergic projections from the DMV to periportal and midlobular regions (Hwang et al., 2024). This discrepancy between immunohistochemical and genetic detection of cholinergic fibers in the mouse, combined with the paucity of human VAChT data, makes it impossible to determine whether the human and rodent cholinergic innervation patterns are fundamentally different or merely differentially detectable with current tools.

Experimental accessibility creates an asymmetry that distorts the literature: rodent studies can employ viral tracing, chemogenetics, optogenetics, time-course analysis, and genetic models that are essentially impossible in humans, generating mechanistic knowledge that cannot be directly validated in the species of ultimate clinical interest.

### 7.2 A Comparative framework and its interpretive implications

Synthesizing the comparative evidence above (**Table 3**), three levels of inference can be distinguished. Conserved features—robust portal-tract sympathetic and sensory innervation, periportal predominance, and vagal regulation of hepatic glucose and bile handling—likely reflect species-general principles and constitute the most defensible basis for translation. Species-specific features—intralobular sympathetic penetration, hepatocyte–nerve appositions, and the detectability of parenchymal cholinergic fibers—are where rodent data most frequently mislead when extrapolated to humans. Methodologically contingent features—including the apparent absence of mouse parenchymal VAChT immunoreactivity—may reflect detection limits rather than true biological difference. We therefore interpret the existing literature as supporting qualitative translation of the periportal-centric, spatially organized model, but not quantitative extrapolation of rodent nerve-density or nerve–cell-contact measurements to the human liver. Each translational claim should be explicitly assigned to one of these three levels to avoid overreach.

### 7.3 Anatomical scaling and lobular organization

The human liver is approximately 2,000 times larger than the mouse liver by mass (~1,500 g vs. ~0.7–1.0 g) and contains roughly 200 times as many hepatic lobules. This scaling has several neuroanatomical consequences.

One cross-species scaling issue concerns conduction distance. A sympathetic postganglionic axon innervating a peripheral hepatocyte in the human liver must travel centimeters from the celiac ganglion to its target, compared to millimeters in the mouse. The metabolic cost of maintaining such long axons, the vulnerability to ischemic and metabolic injury, and the kinetics of neurotransmitter release and reuptake may all differ between species.

A second issue is how nerve density scales with liver size. The absolute number of nerve fibers innervating the liver does not scale linearly with liver mass. The human liver receives proportionally fewer nerve fibers per unit volume than the mouse liver, meaning that the average hepatocyte in the human liver is served by a sparser neural network. This has implications for the dominant mode of neural signaling: if each hepatocyte is unlikely to be directly contacted by a nerve fiber, paracrine volume transmission becomes the default mechanism, and the spatial range of neurotransmitter diffusion becomes a critical functional parameter.

Species differences in lobular architecture further complicate comparison. The classic hexagonal hepatic lobule is not a uniform structure across species. In the human liver, the connective tissue separating adjacent lobules is less distinct than in the pig or camel, and the portal tracts are embedded in a more continuous parenchymal mass. This “non-septate” lobular architecture means that human intralobular nerve fibers are not guided by well-defined connective tissue planes, potentially resulting in less stereotyped branching patterns than in species with more septate lobules (Akiyoshi et al., 1998).

Regional asymmetry represents another translational consideration. The left and right lobes of the human liver receive differential neural input: the left lobe is preferentially innervated by the left vagus nerve and left celiac plexus, while the right lobe receives right-sided input. Whether this produces functional asymmetry in neural regulation (analogous to the well-known functional asymmetry of hepatic lobar metabolism) has not been investigated.

### 7.4 Methodological confounding in rodent studies

Four common experimental approaches in rodent hepatic neurobiology carry interpretative limitations that are often underappreciated.

A major experimental confound is that hepatic branch vagotomy is not liver-specific. The common hepatic branch of the rodent vagus nerve divides into a hepatic branch proper (supplying the liver) and a gastroduodenal branch that supplies the pylorus, proximal duodenum, and pancreas (Berthoud, 2004; Berthoud et al., 2024). Standard hepatic branch vagotomy transects both branches, producing effects on gut hormone secretion (CCK, GLP-1, PYY), pancreatic exocrine and endocrine function, gastroduodenal motility, and intestinal nutrient sensing—any of which can indirectly alter hepatic metabolism independent of lost hepatic vagal innervation.

Viral tracing introduces a different confound because extra-hepatic transduction can occur. AAV injected into the liver parenchyma can leak into the hepatic veins, inferior vena cava, and systemic circulation, transducing neurons in the heart, kidney, gastrointestinal tract, and brain. The resulting labeling of “liver-projecting” neurons may include neurons that were transduced via systemic viral spread rather than by retrograde transport from hepatic axon terminals. Careful controls—including measurement of viral genome copies in distant organs and sham injection into the portal vein—are essential but not uniformly performed.

Chemical denervation methods likewise lack organ specificity. Intraportal 6-hydroxydopamine (6-OHDA) injection depletes hepatic norepinephrine but also produces significant denervation of the kidney, heart, and other organs (Wheatley et al., 1993). Systemic administration of the catecholaminergic neurotoxin 6-OHDA or the cholinergic blocker scopolamine produces widespread autonomic effects that preclude attribution of metabolic changes to hepatic denervation alone.

Similarly, systemic pharmacology cannot isolate hepatic neural contributions. A drug administered systemically that acts on adrenergic or cholinergic receptors will affect every organ expressing those receptors. A reduction in hepatic glucose output following systemic β-blocker administration could reflect direct blockade of hepatocyte β2-adrenoceptors, reduced sympathetic drive to the liver, reduced glucagon secretion, altered splanchnic blood flow, or central nervous system effects—none of which specifically implicate hepatic nerves.

### 7.5 Limitations of human evidence

Human hepatic neuroanatomy is constrained by limitations that do not apply to experimental animal studies.

A first limitation of the human literature concerns sample size and selection. Most human studies of hepatic innervation involve fewer than 30 specimens (Mizuno et al., 2021; Chen et al., 2024). “Normal” human liver tissue is almost never from truly healthy individuals; it typically comes from unaffected margins of hepatic resection specimens (where the primary tumor or metastasis may have altered local and systemic physiology), donor livers rejected for transplantation (which carry intrinsic abnormalities), or autopsy specimens (with variable post-mortem intervals) (Chen et al., 2024; Lee et al., 2025).

A second limitation is tissue quality. Surgical liver biopsies experience variable warm ischemia time between vascular clamping and fixation. Cold ischemia during transport, fixation method (formalin vs. paraformaldehyde), fixation duration, and paraffin embedding all affect antigenicity and antibody penetration. Steatosis—present in a large fraction of available human liver specimens given the prevalence of MASLD—impairs tissue clearing and increases light scattering (Lee et al., 2025).

A third limitation is the absence of dynamic neural activity data. Unlike rodent studies, where calcium imaging, electrophysiology, and chemogenetic manipulation of defined neural populations are possible, human hepatic neurobiology is confined to static structural snapshots. We can describe where nerves are in the human liver, but we cannot directly observe what they do.

Finally, interpretation is complicated by confounding variables. Age, sex, body mass index, medication use (particularly β-blockers, which are common in patients with cirrhosis), disease etiology, disease stage, and time since diagnosis all influence neural structure but are rarely adequately controlled in small human cohorts.

### 7.6 Distinguishing direct human evidence from experimental evidence

To discipline the translational argument, we distinguish direct human evidence from experimental (animal) evidence throughout. Direct human evidence is limited to static anatomical and molecular observations: human tissue imaging (3D/Airyscan and conventional histology), human histological and immunohistochemical analysis, and emerging human spatial mapping (Chen et al., 2024; Lee et al., 2025). Experimental evidence—retrograde tracing, Cre-dependent genetic labeling, chemogenetics, optogenetics, and terminal ablation—provides the bulk of current mechanistic insight but is, with rare exceptions, confined to rodents and cannot currently be performed in humans. Mechanistic conclusions in this review should therefore be read with their evidentiary source in mind: statements about where human nerves are located rest on direct evidence, whereas statements about what those nerves do rely on experimental models and are inferred, not demonstrated, for the human liver.

## 8 A translational roadmap for the human hepatic neural atlas

### 8.1 Establishing a standardized human reference atlas

The first and most urgent need is a standardized, publicly accessible human hepatic neural atlas that maps the 3D distribution of sympathetic, parasympathetic, sensory, and peptidergic nerve fibers across the healthy human liver and across the major disease stages. Such an atlas should include:

A biobank of well-annotated specimens spanning healthy donor liver, simple steatosis, MASH (with fibrosis stages F0–F4), viral hepatitis (pre- and post-SVR), alcoholic liver disease, PSC, PBC, and biliary atresia. Standardized sampling locations: right lobe (segment 5/6 and segment 7/8), left lobe (segment 2/3), and caudate lobe, to assess regional heterogeneity and left–right asymmetry. A standardized multi-marker panel: PGP9.5 (pan-neuronal), TH (sympathetic), VAChT and/or ChAT (cholinergic), CGRP (sensory peptidergic), NF200 (myelinated), plus at least one vascular marker (CD31), biliary marker (CK19), and macrophage marker (CD68) for spatial context. A standardized tissue processing and imaging pipeline: optimized clearing protocol (e.g., photochemical bleaching plus high-refractive-index polymer embedding (Chen et al., 2024; Lee et al., 2025)), LSFM for macroscopic mapping, Airyscan confocal for periportal and lobular regions of interest, and a common computational workflow for nerve segmentation and quantification. Mandatory reporting of metadata: donor/patient age, sex, BMI, disease etiology, fibrosis stage (METAVIR or equivalent), steatosis grade, inflammation grade, medications (particularly β-blockers, anticholinergics, and sympathomimetics), and tissue ischemic time.

A 2026 spatial atlas generated from healthy live-donor human livers provides an important molecular and anatomical reference for this effort and reveals finer human zonation than conventional three-zone models (Yakubovsky et al., 2026). However, its primary focus is hepatic cell-state and gene-expression architecture rather than direct reconstruction of neural fibers. A hepatic neural atlas should therefore build on, rather than duplicate, this resource by registering nerve subtype, trajectory, varicosity, and nerve–cell distance data onto compatible spatial coordinates.

### 8.2 Integrating structure with molecular identity

Structural maps alone cannot establish functional connectivity. The atlas must be integrated with molecular data to address three questions (1): From which neuronal population does a given nerve fiber originate? (2) Which receptors are expressed by cells adjacent to that fiber? (3) Does anatomical proximity correspond to functional signal transmission?

This integration can be achieved through three complementary approaches. First, spatial transcriptomics on the same or serial sections used for 3D nerve imaging can map the expression of neurotransmitter receptors (ADRA1A, ADRB2, CHRM3, HTR3A, P2RX3, TRPV1) and neurotrophins (NGF, BDNF, NTF3) onto the same spatial coordinates as the nerve fiber map. Second, single-nucleus RNA-seq of human nodose ganglia and thoracic DRG—performed on specimens from organ donors—can identify molecularly defined neuronal subtypes and their receptor and neuropeptide expression profiles, enabling inference of which neuronal populations supply which hepatic structures. Third, spatial proximity analysis—quantifying the distance between individual nerve varicosities and immunostained target cells—can generate testable hypotheses about functional connectivity that can be validated in human-compatible functional models (Section 8.3).

A critical principle is that each level of integration—structural, molecular, functional—provides necessary but not sufficient evidence for a functional neuro-effector connection. Only the convergence of all three levels constitutes strong evidence.

### 8.3 Human-compatible functional models

Given the impossibility of functional neural tracing or optogenetic manipulation in living human liver, alternative models must be developed to validate structural predictions.

Human liver precision-cut slices (PCLS) represent one candidate platform. PCLS preserve portal, biliary, and sinusoidal architecture for short-term culture (Olinga and Schuppan, 2013). Electrical field stimulation combined with neurotransmitter antagonists could test whether residual neural elements alter glucose output, bile acid synthesis, or cytokine release; this application remains to be validated.

Ex vivo normothermic machine perfusion (NMP) provides another candidate platform. NMP maintains intact donor-liver physiology before transplantation (Nasralla et al., 2018). Incorporating selective neural or receptor stimulation with perfusate sampling could test acute neuro-hepatic responses, but preservation of functional autonomic terminals during NMP must first be demonstrated.

Neuralized liver organoids and assembloids represent a longer-term experimental direction. Building on vascularized human liver organoid systems (Takebe et al., 2013), hepatic organoids could be co-cultured with pluripotent-stem-cell-derived autonomic or sensory neurons. Compartmentalized microfluidic systems would permit selective axonal stimulation and measurement of hepatic responses; such liver–neuron assembloids remain an emerging model rather than an established platform.

Finally, donor–recipient paired analysis in liver transplantation offers a natural denervation–reinnervation model. Procurement interrupts extrinsic hepatic innervation, and available human studies indicate absent early sympathetic reinnervation but partial perivascular nerve regeneration over longer follow-up (Kjaer et al., 1994; Hanabata et al., 2026). Paired donor and recipient biopsies from the same graft, combined with metabolic phenotyping, could help distinguish structural reinnervation from functional recovery.

### 8.4 From neural architecture to clinical applications

The hepatic neural framework developed here carries several potential clinical implications, which we rank by their proximity to current practice. Neural biomarkers are the nearest application: nerve fiber density (NFAR), nerve-to-cell proximity, and the proportion of degenerating versus sprouting fibers could serve as quantitative descriptors of the neural niche, potentially stratifying patients by disease stage or treatment response, although their independent prognostic value remains to be established. Disease classification could incorporate neural remodeling as a complementary axis to conventional inflammation and fibrosis scoring, and sex-stratified neural metrics may refine risk assessment in MASLD. Precision medicine could, in principle, use spatial neural maps to predict which patients will benefit from anti-adrenergic or anti-fibrotic therapy and to monitor neural recovery after fibrosis regression. Neuromodulation strategies—whether vagal, sympathetic, or sensory afferent—remain preclinical, and their clinical translation depends on the human atlas and functional models described above. These applications are, at present, hypotheses to be tested rather than established clinical tools.

### 8.5 Toward spatially precise neuromodulation

While the clinical application of liver-directed neuromodulation remains aspirational, the spatial framework developed in this review suggests several rational targets, which we categorize below as established pharmacological approaches, mechanistically plausible but preclinical strategies, and highly speculative interventions:

Hepatic branch vagus nerve stimulation (hbVNS) remains a preclinical, mechanistically plausible strategy rather than an established liver-selective therapy. Systemic inflammatory-reflex studies show that vagal stimulation can suppress inflammatory responses (Borovikova et al., 2000; Tracey, 2002), but they do not establish direct hepatic-branch control of Kupffer cells or glucose metabolism. The common hepatic branch is anatomically difficult to isolate, and selective stimulation of hepatic versus gastroduodenal fibers has not been achieved in humans.

Sensory afferent receptor targeting—for example, TRPV1 or CCKAR agonists delivered to the portal region—could modulate hepatic interoceptive signaling without requiring direct nerve stimulation.

Liver-specific drug delivery using nanoparticle formulations, adeno-associated viral vectors with hepatotropic serotypes, or portal vein infusion could target neural signaling while minimizing systemic off-target effects.

Adrenergic and neuropeptide receptor modulation—carvedilol, prazosin, and other adrenergic blockers shown to reduce fibrosis in preclinical models (Medina Pizaño et al., 2023)—represent the most immediately translatable pharmacological approach, though their effects are not liver-specific.

Image-guided neuromodulation using focused ultrasound or magnetic stimulation to non-invasively modulate hepatic nerve activity remains entirely speculative.

It is essential to emphasize that all neuromodulatory approaches targeting the liver are currently at the preclinical stage, and most have only been tested in rodent models of limited human relevance (Section 7). The translational roadmap outlined here is designed to provide the structural and functional foundation without which rational neuromodulation cannot proceed.

## 9 Conclusions

This review leads to three principal conclusions.

First, hepatic innervation is fundamentally spatial. Nerve fibers are not uniformly distributed throughout the liver but are organized into a hierarchy of neurovascular–cellular niches along hilum-to-periphery and portal-to-central gradients. The periportal region, by virtue of its privileged access to portal blood and its dense convergence of sympathetic, parasympathetic, and sensory fibers, emerges as a candidate dominant anatomical integration site for interoception—a site where metabolic, mechanical, and inflammatory signals are detected, integrated, and transmitted to the brain, alongside contributions from the capsule and sinusoidal compartments. The periportal lamellar complex, recently identified by 3D imaging, may serve as a structural scaffold that organizes this neural architecture during development and guides nerve remodeling during fibrosis.

Second, 3D imaging technologies are transforming the study of hepatic innervation from a binary question—”are there nerves in the liver?”—to a quantitative, spatially resolved investigation of where nerves are located, which cells they contact, and how these contacts change in disease. The integration of tissue clearing, volumetric imaging, neural tracing, and spatial omics provides a methodological framework for building a comprehensive human hepatic neural atlas, though substantial technical challenges remain, particularly for human tissue.

Third, the gap between rodent and human hepatic neurobiology is not merely quantitative but qualitative, reflecting species-specific differences in intralobular nerve penetration, hepatocyte–nerve contacts, and cholinergic innervation patterns, compounded by methodological confounds in rodent studies and severe limitations in human tissue access and quality. Bridging this gap will require a concerted effort to generate standardized human structural data, integrate it with molecular identity, and validate functional predictions in human-compatible model systems.

We propose that circulating hormonal and long-loop autonomic regulation should be considered together with, rather than replaced by, a spatially organized local neural network. On this view, the liver is a spatially organized neural organ, and the hepatic neural niche is a useful—but still provisional—framework for understanding how the brain and liver communicate, how this communication fails in disease, and how it might be therapeutically restored.
